# Comparative transcriptome analysis and identification of candidate effectors in two related rust species (*Gymnosporangium yamadae* and *Gymnosporangium asiaticum*)

**DOI:** 10.1186/s12864-017-4059-x

**Published:** 2017-08-23

**Authors:** Si-Qi Tao, Bin Cao, Cheng-Ming Tian, Ying-Mei Liang

**Affiliations:** 1 The Key Laboratory for Silviculture and Conservation of Ministry of Education, Beijing Forestry University, Beijing, 100083 China; 2 Museum of Beijing Forestry University, Beijing, 100083 China

**Keywords:** Comparative transcriptome, Rust fungi, RNA-Seq, Orthologous gene, Candidate effectors, Divergence time

## Abstract

**Background:**

Rust fungi constitute the largest group of plant fungal pathogens. However, a paucity of data, including genomic sequences, transcriptome sequences, and associated molecular markers, hinders the development of inhibitory compounds and prevents their analysis from an evolutionary perspective. *Gymnosporangium yamadae* and *G. asiaticum* are two closely related rust fungal species, which are ecologically and economically important pathogens that cause apple rust and pear rust, respectively, proved to be devastating to orchards. In this study, we investigated the transcriptomes of these two *Gymnosporangium* species during the telial stage of their lifecycles. The aim of this study was to understand the evolutionary patterns of these two related fungi and to identify genes that developed by selection.

**Results:**

The transcriptomes of *G. yamadae* and *G. asiaticum* were generated from a mixture of RNA from three biological replicates of each species. We obtained 49,318 and 54,742 transcripts, with N50 values of 1957 and 1664, for *G. yamadae* and *G. asiaticum*, respectively. We also identified a repertoire of candidate effectors and other gene families associated with pathogenicity. A total of 4947 pairs of putative orthologues between the two species were identified. Estimation of the non-synonymous/synonymous substitution rate ratios for these orthologues identified 116 pairs with Ka/Ks values greater than1 that are under positive selection and 170 pairs with Ka/Ks values of 1 that are under neutral selection, whereas the remaining 4661 genes are subjected to purifying selection. We estimate that the divergence time between the two species is approximately 5.2 Mya.

**Conclusion:**

This study constitutes a de novo assembly and comparative analysis between the transcriptomes of the two rust species *G. yamadae* and *G. asiaticum*. The results identified several orthologous genes, and many expressed genes were identified by annotation. Our analysis of Ka/Ks ratios identified orthologous genes subjected to positive or purifying selection. An evolutionary analysis of these two species provided a relatively precise divergence time. Overall, the information obtained in this study increases the genetic resources available for research on the genetic diversity of the *Gymnosporangium* genus.

**Electronic supplementary material:**

The online version of this article (doi:10.1186/s12864-017-4059-x) contains supplementary material, which is available to authorized users.

## Background

*Gymnosporangium* species are mainly distributed in the Northern Hemisphere and are mostly demicyclic, whereby two hosts are required for the pathogen to complete its disease cycle [1]. *Gymnosporangium* rusts are unique in that the telial host is a gymnosperm, whereas the spermogonia and aecial hosts are dicotyledonous plants, especially the Pomoideae of the Rosaceae [2]. *G. yamadae* Miyabe ex Yamada and *G. asiaticum* Miyabe ex Yamada are difficult to distinguish in the wild, and both are economically important species for apple and pear production and for cultivated Junipers in Asia.

**Fig. 1:**
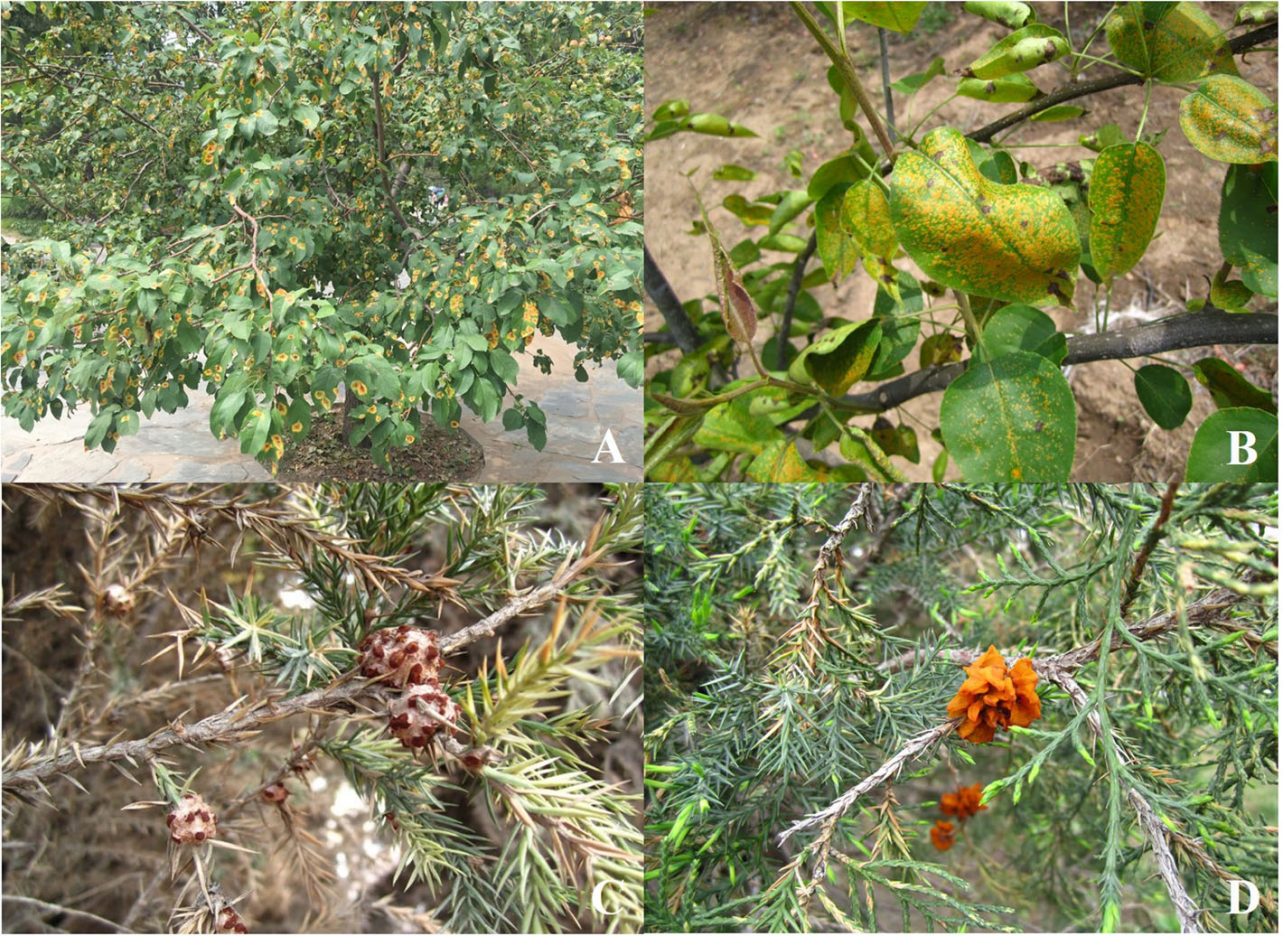
Rust disease symptoms on apple (**a**) and pear (**b**) leaves

The production of apples and pears in most areas of northern China accounts for more than half of the world’s output and thus plays an essential strategic role in export, agricultural structure adjustment, and farmer income [3, 4]. However, the growth of harmful organisms has also shown an annually increasing trend. Rust diseases caused by *Gymnosporangium* species on apple and pear trees (Fig. 1a and b), which commonly occur in many provinces, threaten economic orchard development [5, 6]. Similarly, as landscape trees, junipers have been widely planted in places such as parks, districts, and graveyards; however, the strange appearance and bright colour of the telial horns of *Juniperus* plants has caused much concern because their aesthetic value is impacted by rust fungi (Fig. 1c and d). Rust fungal infections cause stem swelling and deformed branches, occasionally resulting in branch knots that can kill the tree hosts.

**Fig. 2:**
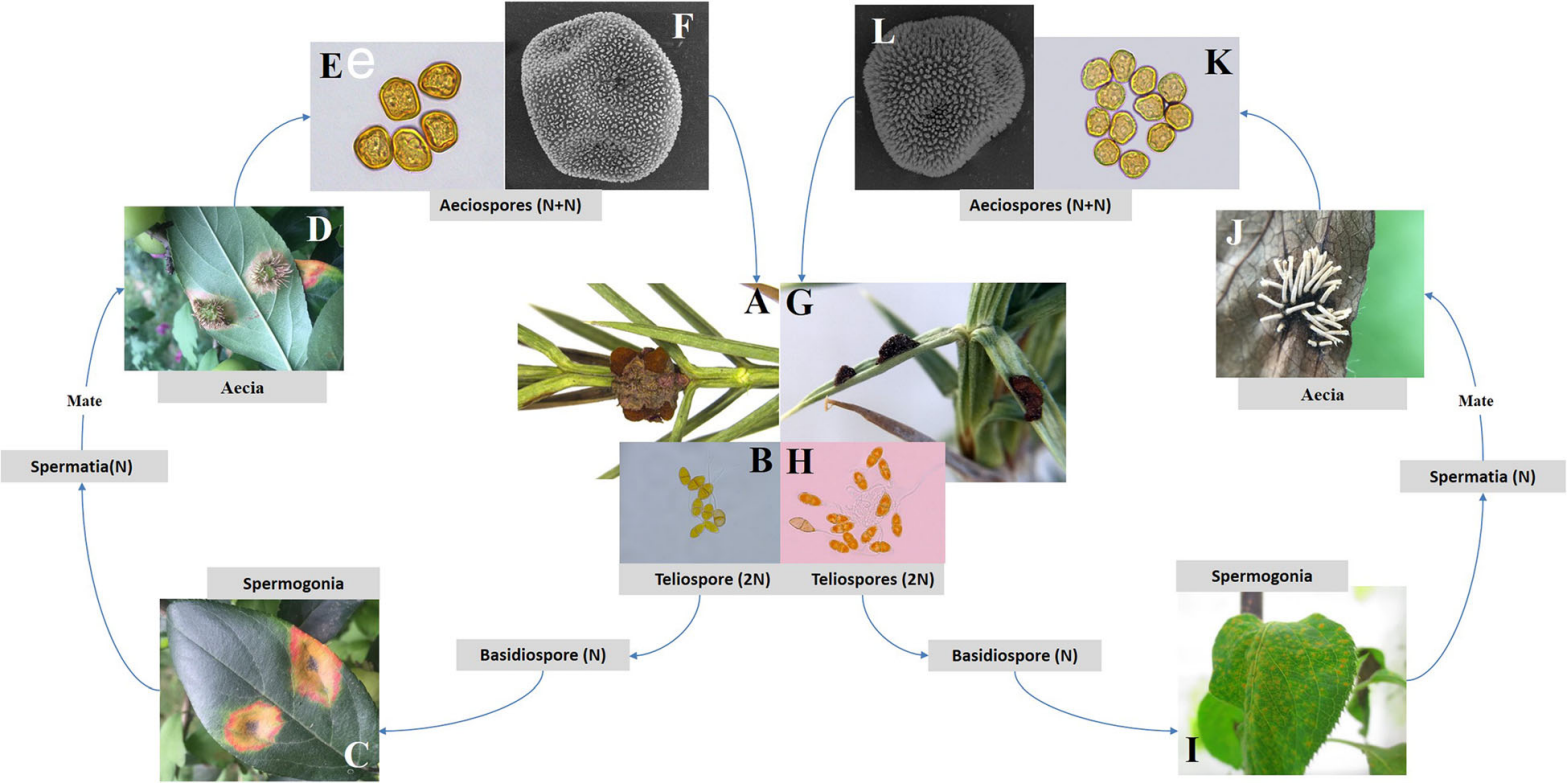
**a**-**f** Disease cycle of *G. yamadae*. **a** Telia on a Juniper branch gall. **b** Teliospores observed with a light microscope. **c** Spermogonia on the upper surface of an apple leaf; **d** Tubular aecia on the lower surface of a leaf. **e** and **f** Aeciospores observed with a light microscope and scanning electron microscope, respectively. **g**-**l** Disease cycle of *G. asiaticum.* **g** Cushion-shaped telia on the leaves of juniper; **h** Teliospores observed with a light microscope. **i** Spermogonia on the upper surface of a pear leaf. **j** Tubular aecia on the lower surface of a leaf. **k** and **l** Aeciospores observed with a light microscope and scanning electron microscope, respectively

Although the two species share juniper trees as a host during their telial stage*, G. yamadae* overwinters in branch galls in the form of mycelia, and *G. asiaticum* overwinters in stem swelling or needle lesions. Usually, after a heavy spring rain, the telial horns extrude from the branch galls, stem swelling, or needle lesions on the host juniper trees. Teliospores in the gelatinous horns produce basidiospores that are wind-blown to Maloideae hosts. If infection is successful in the Maloideae hosts, spermogonia develop in orange lesions on the upper surfaces of leaves. After a period of time, aecia also develop in the same orange lesions but on the lower surfaces of the leaves. Mature aeciospores (spores produced in the aecia) are wind-blown to the juniper trees in the same year from early summer to fall. If infection is successful in the juniper host, galls, witches’ brooms, or stem swellings form, and the telial horns grow from these symptomatic tissues the following spring or in subsequent years (Fig. 2).

Most previous researchers focused on morphological observations and phylogenetics among these species [1, 2]. Research has been hampered by the fact that few *Gymnosporangium* genomic resources are available. Before our current study, a single report in 2014 suggested that *G. confusum* has the largest fungal genome [7]. A paucity of genetic data such as genome sequences, transcriptome sequences, and associated molecular markers have made the control of rust diseases resulting from the *Gymnosporangium* a challenging task.

Transcriptome analysis has proven to be a very efficient approach for the discovery of candidate genes, the annotation of genes and the development of PCR-based molecular markers when a complete genome sequence is lacking. The first obligate plant biotrophic pathogens of the Basidiomycota phylum to be sequenced were the poplar leaf rust pathogen *Melampsora larici-populina* and the wheat stem rust pathogen *P. graminis* f. sp. *tritici*, and this sequencing was performed in 2011 using the Sanger whole-genome shotgun strategy [8]. This great genomic-level achievement has allowed the design of custom whole-genome oligoarrays for conducting genome-wide expression surveys during the infection process of rust species on their telial hosts (poplar and wheat, respectively) [8, 9]. With the rapid development of next-generation sequencing, RNA-sequencing (RNA-Seq) has become an instrumental method for the analysis of multiple aspects of fungal transcriptomes, such as transcriptional profiling, putative virulence gene identification, secretome analysis, gene model analysis, alternative transcript splicing, and structural gene annotation [10]. Since 2011, RNA-Seq technology has been used to increase the understanding of dynamic plant-pathogen interactions and for pathogen transcript profiling during the infection process of several rust fungi. For the sequenced rust fungus *M. larici-populina*, infected poplar at 18, 24 and 48 h post-infection was subjected to 454 pyrosequencing, which yielded an assembly of 90,398 contigs. However, due to the difficulty of isolating rust RNA from the host, only a small fraction of this assembly, consisting of 649 contigs, was assigned to the genome reference sequence [11]. Furthermore, for some non-model rust fungi for which genomic information is lacking, RNA-Seq has been used to compare different stages of infection to reveal patterns of gene expression during the infection process. For example, a total of 27,715 transcripts, including 19,000 novel transcripts, were obtained from *Phakopsora pachyrhizi*-infected *Glycine max* leaves at four time points, providing insight into molecular events and their timing throughout the lifecycle of *P. pachyrhizi* [12]. Similarly, large-scale sequencing of a mixture of RNA from *Hemileia vastatrix* and infected *Coffea arabica* resulted in 22,774 assembled contigs and identified several novel fungal genes and candidate effectors [13]. De novo sequencing of the *Cronartium ribicola* transcriptome was performed during its four lifecycle stages, and the analysis predicted 734 unique proteins and characterized some candidate effectors and other pathogenicity determinants. Moreover, this study identified differentially expressed genes during different infection stages to gain a clear understanding of molecular interactions based on comprehensive transcriptome profiling [10]. Genome-wide expression studies of Pucciniales species have revealed that the genetic programme expressed by rust fungi is finely regulated [14]. Massive genomic resources, including unique genes and putative candidate effectors with unknown functions to be identified in future work, are now available for this fungal order. An alternative application of RNA-Seq is the isolation of RNA from purified haustoria to establish the transcriptome profile, focusing on the key rust determinants involved in nutrient acquisition and the delivery of effectors to host cells [15–17].

RNA-Seq technology, which is more efficient and less expensive, has also been applied in comparative transcriptional analyses of closely related species because it provides additional genomic resources, such as specific simple sequence repeat (SSR) primers or single-copy nuclear gene primers [18]. RNA-Seq also provides information regarding the processes of speciation and adaptive evolution, including the ability to identify divergent and conserved genes and to estimate the divergence time [19, 20].

Our collection survey showed that *G. yamadae* and *G. asiaticum* can parasitize different parts of the same cypress tree (*Juniperus chinensis* L.). *G. yamadae* produces tongue-shaped telia on globoid swellings or galls; however, the telia of *G. asiaticum* form wedge- or pulvinate-shaped structures on green stems and do not induce hypertrophy (Fig. 2). After performing de novo sequencing of the transcriptomes of *G. yamadae* and *G. asiaticum* using RNA-Seq during the telial stage of their lifecycles, we expected to 1) identify the transcriptomes of *G. yamadae* and *G. asiaticum*, increasing the genetic resources available for research on the diversity of the *Gymnosporangium* genus, 2) identify the secretome and putative candidate effectors in these two species for future functional analysis of virulence and pathogenicity, 3) identify homologous genes between these two species through the calculation of Ka/Ks ratios to determine whether selective pressure acts on these protein-coding genes, and 4) determine the evolutionary kinetics of these two species, including an estimation of the divergence time between these species.

## Results

### De novo assembly of the *G. yamadae* and *G. asiaticum* transcriptomes

In our study, we built transcriptomes of *G. yamadae* and *G. asiaticum* during the telial stage of their lifecycles. About one month after the teliospores began to extrude from galls macroscopically, three biological replicates were collected for each species. And approximately 50-mg sample of teliospores from each replicate was used for total RNA extraction and the cDNA libraries were then independently constructed and subsequently sequenced using an Illumina Hi-Seq 2000 platform (ca. 150 million 100-bp paired end (PE) raw reads were generated from each of the three cDNA libraries for *G. yamadae* and *G. asiaticum*; Table 1). These data have been deposited in the NCBI Sequence Read Archive. After removing adapters and low Phred quality sequences, approximately 150 and 160 million high-quality sequence reads were obtained from the three *G. yamadae* and *G. asiaticum* libraries, respectively (Table 1). The trimmed reads were then assembled into transcriptomes using the Trinity package [21]. To test the overall assembly quality, the clean reads were mapped against the transcriptome and read count for each unigenes was obtained from the mapping results through RNA-Seq using Expectation Maximization (RSEM) software [22]. The average numbers of mapped reads for *G. yamadae* and *G. asiaticum* were 43,235,275 (83.98%) and 42,550,674 (78.32%), respectively (Table 1). To quantify gene expression abundance, FPKM (fragment per kilobase per transcript per million mapped reads) was calculated for each sample by converting the read count value of all unigenes, meantime, the FPKM density distribution profiles were constructed to reflect the gene expression pattern of each sample (Additional file 1). More than half of the unigenes with PFKM value more than 0.3 were regarded as expressed and retained in each sample of *G. yamadae* and *G. asiaticum*, respectively (Table 1, Additional file 1).

**Table 1:** Statistics of the RNA-Seq data

Samples	Raw reads	Clean reads	Q20 (%)	Q30 (%)	Mapped reads	Expressed unigenes (FPKM > 0.3)
DSXGY_1	47,629,874	4,6917,624	97.18	93.82	38,545,026 (82.29%)	18,619 (53.04%)
DSXGY_2	59,428,164	58,748,388	96.52	91.47	50,102,470 (85.29%)	16,847 (47.99%)
DSXGY_3	49,485,212	48,752,948	97.34	93.42	41,058,330 (84.35%)	18,561 (52.88%)
DSXGA_1	45,287,548	44,616,412	97.01	93.39	33,171,198 (74.36%)	16,406 (45.14%)
DSXGA_2	52,290,920	51,638,726	96.41	91.24	39,759,730 (77.01%)	18,860 (51.89%)
DSXGA_3	66,334,424	65,471,582	96.21	90.78	54,721,096 (83.59%)	18,000 (49.53%)

DSXGY_1–3 and DSXGA_1–3 represent independent biological replicates of *G. yamadae* and *G. asiaticum*, respectively. Q20: Percentage of bases with a Phred value >20; Q30: Percentage of bases with a Phred value >30; and Mapped reads: Number and percentage of reads from each date of data trimming that were mapped back onto the assembled transcriptome

We obtained 49,318 transcripts, with a mean length of 1006 bp and an N50 value of 1957, for *G. yamadae*, and 54,742 transcripts, with a mean length of 1059 bp and an N50 value of 1664, for *G. asiaticum* (Table 2 and Additional file 2). Using overlapping information from high-quality reads, we identified 35,102 and 36,343 unigenes for *G. yamadae* and *G. asiaticum*, with average lengths of 756 and 754 bp, respectively (Table 2 and Additional file 3).

**Table 2:** Summary of transcripts and unigenes of *G. yamadae* and *G. asiaticum* assembled using Trinity software

	*G. yamadae*	*G. asiaticum*
Transcripts	49,318	54,742
Max length	19,126	16,773
Average length	1006	1059
N50	1957	2070
Total residues	49,601,912	57,968,265
Unigenes	35,102	36,343
Max length	19,126	16,773
Average length	756	754
N50	1654	1664
Total residues	26,521,164	27,406,256
Full-length CDS	18,467	17,769
Mean length	848	724
> 90 bp	18,303 (99.1%)	17,685 (99.5%)

**Fig. 3:**
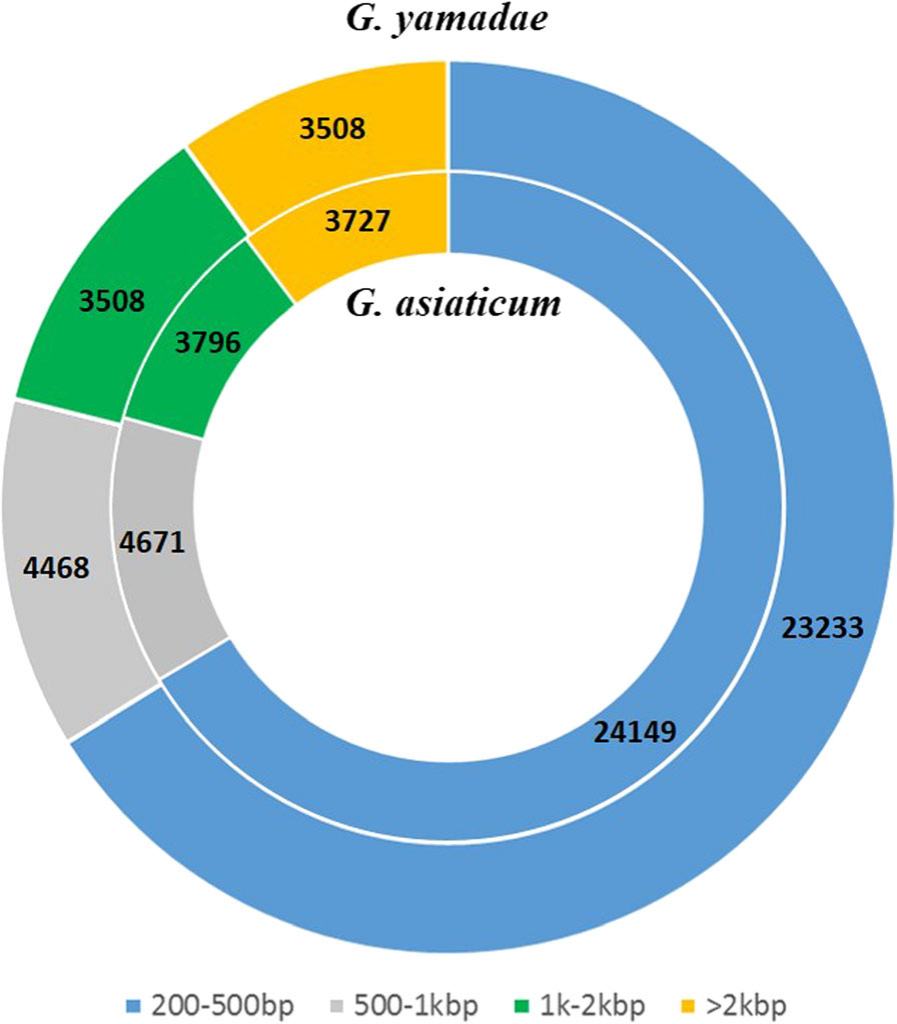
Length distribution of the assembled unigenes of *G. yamadae* (GYY) and *G. asiaticum* (GYA). The outer and inner cycles represent the distribution of GYY and GYA unigenes, respectively. The data in each region indicate the number of unigenes within that range of nucleotide length

Unigenes with lengths between 200 and 500 bp were overrepresented, consisting of approximately 66% of the total number of unigenes for both *G. yamadae* and *G. asiaticum*. The second most abundant class contained unigenes from 500 to 1000 bp, constituting approximately 12.7 and 12.9% of the total number of unigenes for *G. yamadae* and *G. asiaticum*, respectively. The length distribution of unigenes is shown in Fig. 3.

**Fig. 4:**
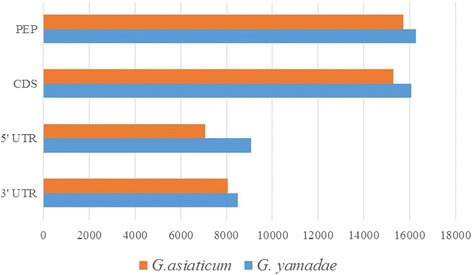
CDS_UTR analysis of unigenes in *G. yamadae* and *G. asiaticum*. The *orange* and *blue bands* represent the number of unigenes of *G. asiaticum* and *G. yamadae*, respectively, containing a 5’UTR, 3’UTR, CDS and PEP. CDS: coding sequence; PEP: amino acid sequence

The coding sequences (CDS) [23] for all unigenes were predicted using ESTScan or Basic Local Alignment Search Tool(BLAST) with the NCBI non-redundant protein sequence (Nr) database and the SwissProt protein database based on an E-value of e^−5^ (see Additional file 4). A CDS longer than 90 bp is approximately 99% identical in both *Gymnosporangium* species. The full-length CDS sequences obtained by BLAST analysis were screened, and the upstream and downstream sequences beyond the CDS as the 5′ untranslated region (UTR) and 3′ UTR sequences were identified, revealing more unigenes with 5′ or 3′ UTRs in *G. yamadae* than in *G. asiaticum* (Fig. 4).

### Functional annotation of the *G. yamadae* and *G. asiaticum* transcriptomes

**Fig. 5:**
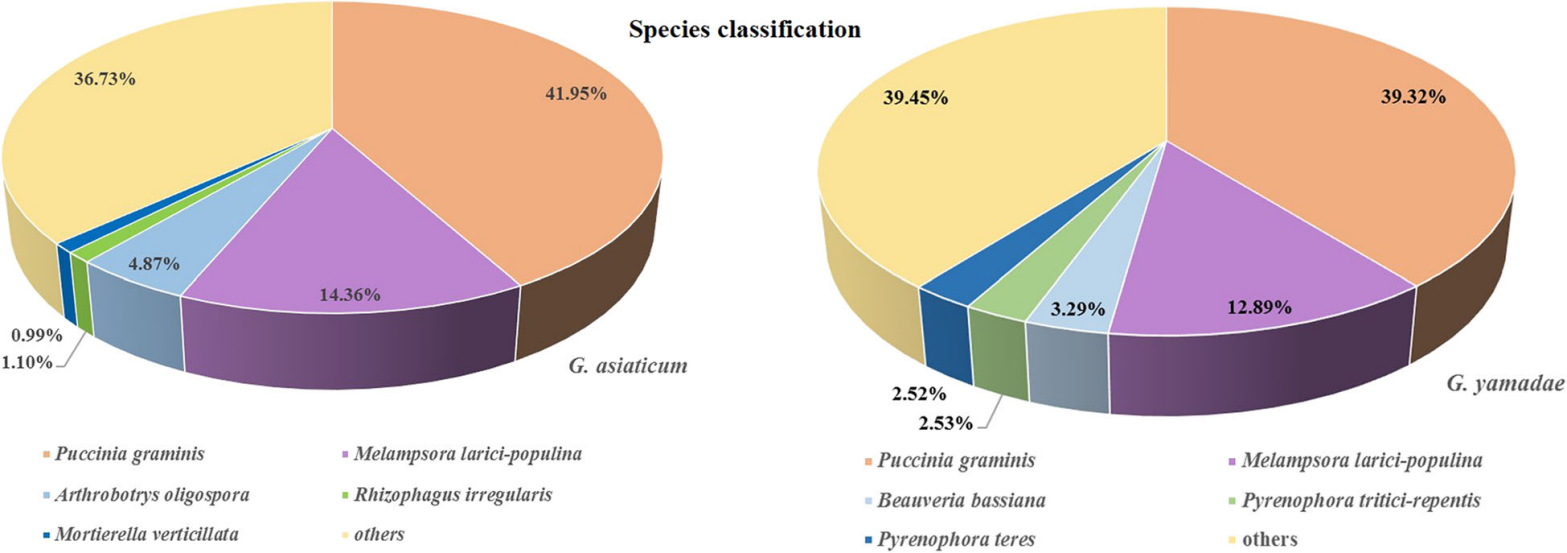
Top-hit species classification for sequences from the two *Gymnosporangium* species subjected to BLASTX analysis against the NCBI-Nr database. Left, *G. asiaticum*; Right, *G. yamadae*

Transcriptome annotation provides insight into the structural, functional, and biological processes in which assembled unigenes are involved [24]. The unigenes of the two transcriptomes were annotated using NCBI BLAST software, and sequence similarity searches were conducted using seven databases: Nr, nucleotide sequences (Nt), protein family (Pfam), eukaryotic orthologue (KOG), SwissProt, Gene Ontology (GO), and Kyoto Encyclopaedia of Genes and Genomes (KEGG) [25–27]. Ultimately, among the 35,102 and 36,343 assembled unigenes, a total of 22,760 (64.83%) unigenes of *G. yamadae* and 21,164 (58.23%) unigenes of *G. asiaticum* were annotated in at least one database, and 2221 (6.32%) and 2699 (7.42%) unigenes, respectively, were annotated in all of the above databases (Table 3 and Additional file 5). Using the Nr database, 14,117 (40.21%) and 15,159 (41.71%) unigenes of *G. yamadae* and *G. asiaticum*, were respectively matched. However, a large proportion of the unigenes (35.17 and 41.77%, respectively) could not be identified, which is a common finding of de novo sequencing studies [28]. For example, in the spruce dwarf mistletoe, *Arceuthobium sichuanense*, transcriptome, approximately 55.42% of the unigenes could not be identified. Our results showed that for *G. yamadae* and *G. asiaticum*, most of the top BLAST hits corresponded to *P. graminis* f. sp. *tritici* (39.3 and 41.9% for *G. yamadae* and *G. asiaticum*, respectively), and the second closest species was another rust fungus, *M. larici-populina* (12.9 and 14.4% for *G. yamadae* and *G. asiaticum*, respectively; Fig. 5).

**Table 3:** Summary statistics of the annotation results

	*G. yamadae*	*G. asiaticum*
Annotated in Nr	14,117 (40.21%)	15,159 (41.71%)
Annotated in Nt	12,273 (34.96%)	9830 (27.04%)
Annotated in KO	5594 (15.93%)	6183 (17.01%)
Annotated in SwissProt	13,385 (38.13%)	12,301 (33.84%)
Annotated in Pfam	12,180 (34.69%)	12,740 (35.05%)
Annotated in GO	13,080 (37.26%)	13,636 (37.52%)
Annotated in KOG	7642 (21.77%)	8002 (22.01%)
Annotated in all Databases	2221 (6.32%)	2699 (7.42%)
Annotated in at least one Database	22,760 (64.83%)	21,164 (58.23%)
Total Unigenes	35,102 (100%)	36,343 (100%)

**Fig. 6:**
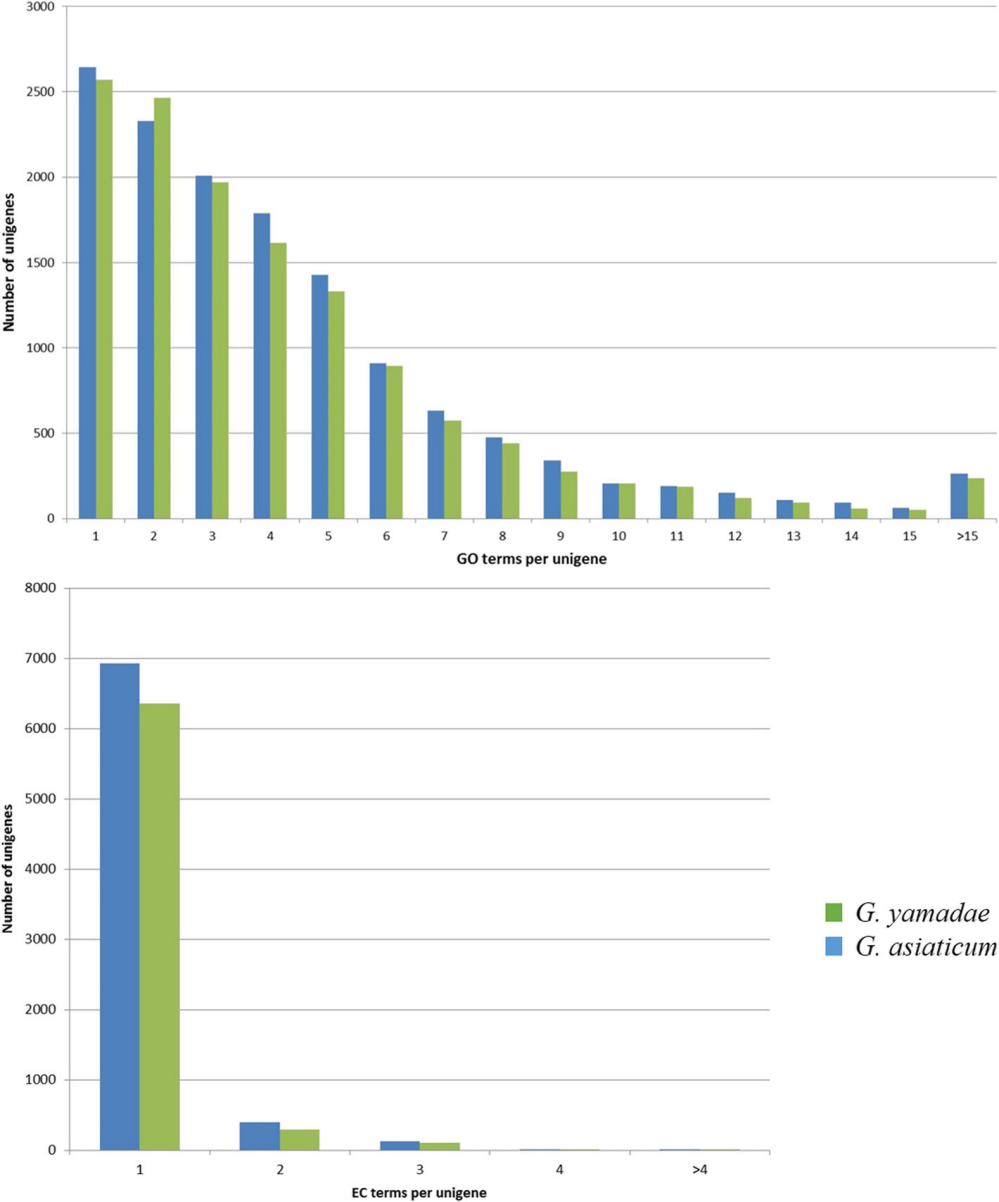
Distribution of the number of GO terms per unigene (*top*) and number of EC terms per unigene (*bottom*) in *G. yamadae* and *G. asiaticum*

To determine the functional annotation of *G. yamadae* and *G. asiaticum* genes, we used the GO database. A total of 13,080 unigenes of *G. yamadae* and 13,636 unigenes of *G. asiaticum* were annotated based on GO terms. Most of the unigenes produced one to seven GO terms (Fig. 6) when annotated to the EC number [29]; the number of annotated unigenes of *G. yamadae* and *G. asiaticum* were 6756 and 7459, respectively, and approximately 90% had only one EC number (Fig. 6). GO terms can be compiled in a graph and are classified by level [18]. Level 1 represents the most general categories and provides the most coverage; as the level number increases, the GO terms provide more specific information and less coverage [30]. In general, level 1 provides a comprehensive description of a process, whereas higher levels provide more specific descriptions.

**Fig. 7:**
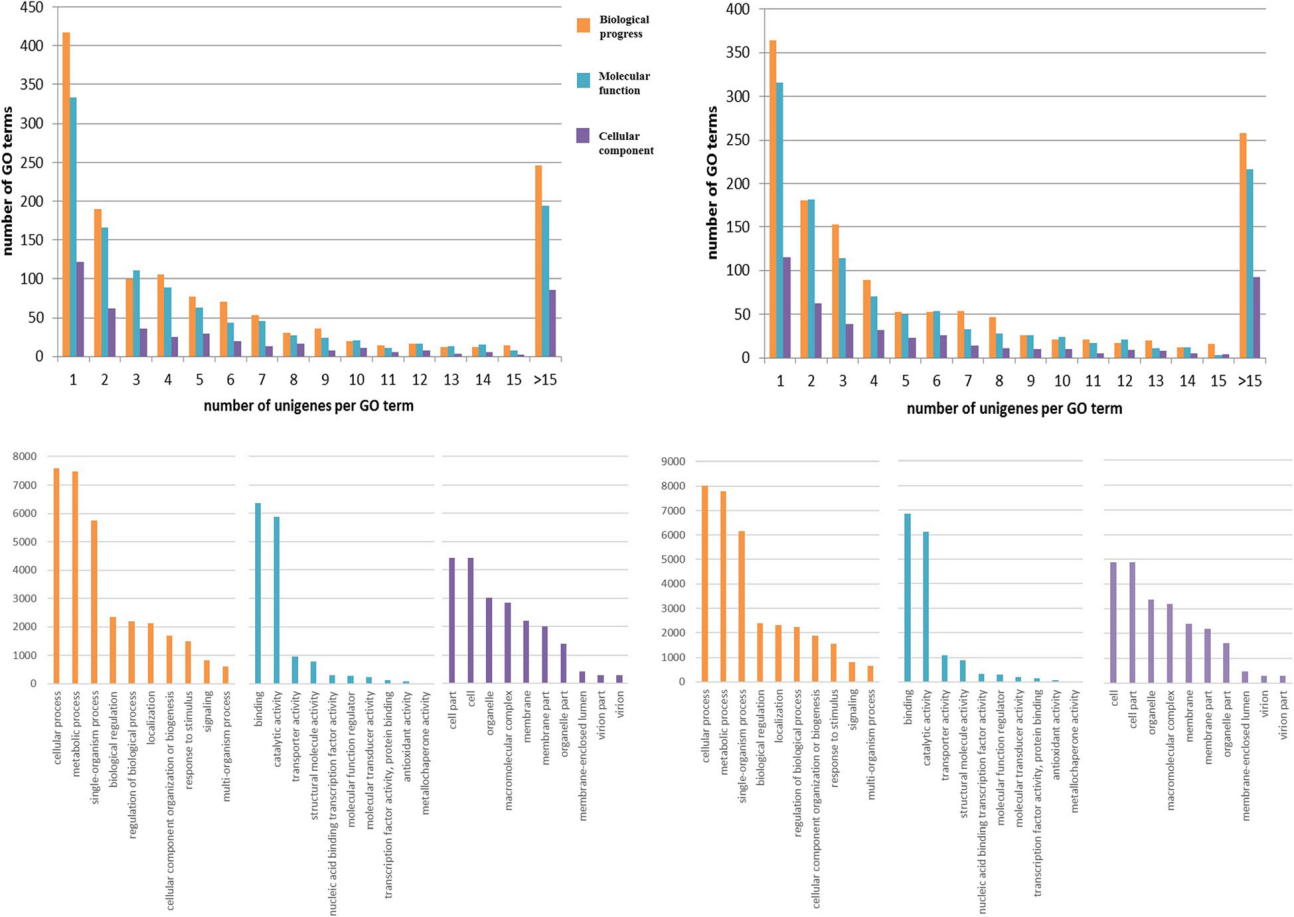
GO term distribution (*top*) and category annotation (*bottom*) for *G. yamadae* (*left*) and *G. asiaticum* (*right*) unigenes. *Top*, numbers of unigenes per GO term in the following functional categories: biological process, molecular function and cellular component. *Bottom*, descriptions of the most abundant gene product for each functional GO category (biological process, molecular function and cellular component)

The majority of GO terms (67.1% for *G. yamadae* and 66.9% for *G. asiaticum*) were related to biological processes (Fig. 7), and of these, cellular, metabolic, and single-organism processes were the three most common GO terms. The cellular component category was assigned to 23.3% of the genes of both *Gymnosporangium* species, and the remaining 9.6 and 9.8% of the annotated unigenes were classified according to the molecular function GO term and were mostly responsible for protein binding, oxidoreductase activity, and hydrolase activity (see Additional file 5).

**Fig. 8:**
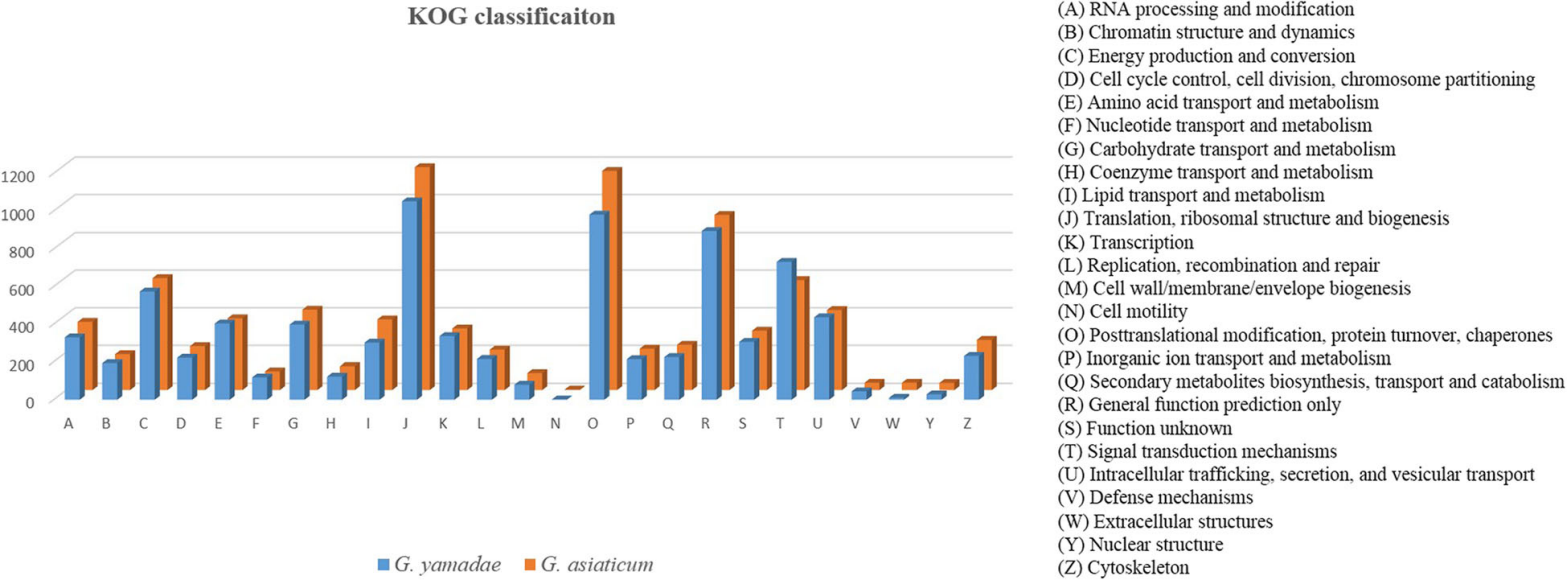
KOG classifications of *G. yamadae* and *G. asiaticum*

To classify the potential functions of these genes, all unigenes were annotated in the KOG database. Orthologous genes have the same function, so the functional annotation can be applied directly to other members of the same COG/KOG cluster. In total, 7642 (21.77%) unigenes of *G. yamadae* and 8002 (22.01%) unigenes of *G. asiaticum* were annotated into 25 KOG classifications (Fig. 8). Among the 25 categories, the cluster of translation, ribosomal structure, and biogenesis unigenes represented the largest group: 1052 (13.77%) unigenes of *G. yamadae* and 1182 (14.77%) unigenes of *G. asiaticum*. The second largest cluster comprised 981 (12.83%) and 1162 (14.52%) unigenes assigned to posttranslational modification, protein turnover, and chaperones, followed by general function prediction only, which accounted for 225 (2.94%) and 239 (2.99%) genes of *G. yamadae* and *G. asiaticum*, respectively (see Additional file 5).

**Fig. 9:**
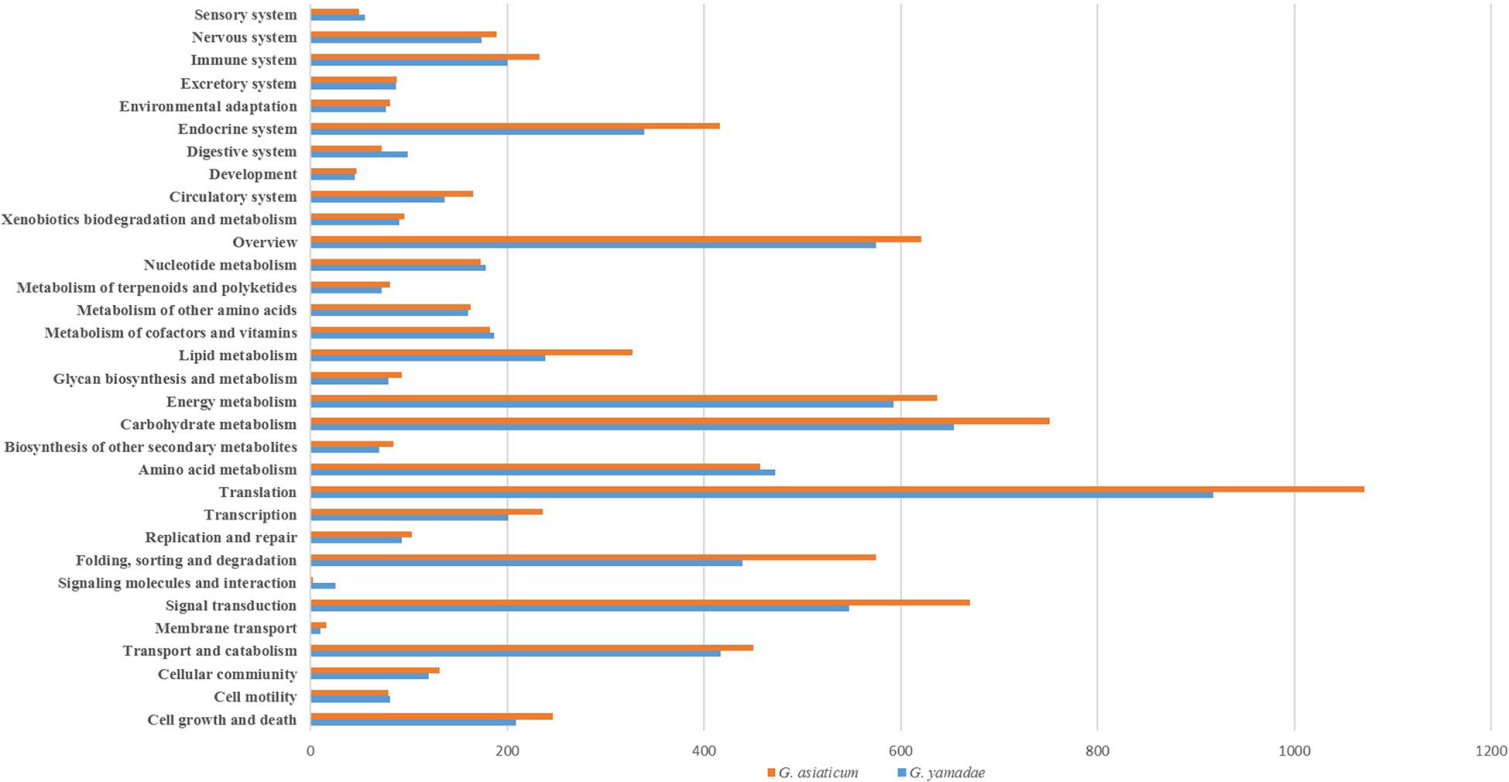
Comparison of the KEGG pathway distribution for the two *Gymnosporangium* species

The annotated unigenes were analysed by BLAST using the KEGG pathway database [31] with KEGG Automatic Annotation Server (KAAS) software. A total of 5594 (15.93%) *G. yamadae* unigenes were assigned to 254 KEGG pathways. The three most enriched pathways were the ribosome pathway (608 unigenes, ko03010), carbon metabolism pathway (367 unigenes, ko01200), and biosynthesis of amino acids pathway (334 unigenes, ko01230). Because *G. asiaticum*, 6183 (17.01%) unigenes were assigned to 254 KEGG pathways, the most enriched pathways are the ribosome pathway (700 unigenes, ko03010), carbon metabolism pathway (388 unigenes, ko01200), and the oxidative phosphorylation pathway (333 unigenes, ko00190; Fig. 9 and Additional file 5).

We performed dbCAN v3.0 HMM-based CAZy annotation [32] to identify CAZy-like proteins in the *G. yamadae* and *G. asiaticum* proteomes. A total of 366 CAZy-like proteins were assigned to 111 CAZy families in *G. yamadae*, and among these annotated carbohydrate-active enzymes (CAZymes), 39.9, 21.6, 19.9, 7.3, 7.1 and 4% were grouped into 42 families of glycosyl hydrolases (GHs), 33 families of glycosyl transferases (GTs), eight families of carbohydrate esterases (CEs), 15 families of carbohydrate binding modules (CBMs), seven families of redox enzymes that act in conjunction with CAZymes (AAs) and four families of polysaccharide lyases (PLs), respectively (Additional file 5). Similarly, in the *G. asiaticum* proteome, 390 CAZy-like annotated proteins were assigned to 111 CAZy families, and 38.2, 24.4, 18.7, 7.4, 6.4 and 4.1% of these were grouped into 43 families of GHs, 34 families of GTs, nine families of CEs, 13 families of CBMs, eight families of AAs and four families of PLs, respectively (Additional file 5).

We also used OrthoMCL [33] to perform a BLAST search of *G. yamadae* and *G. asiaticum* proteins against OrthoMCL database proteins with a cut-off of e^−5^ and a 50% match. The OrthoMCL analysis assigned putative *G. yamadae* and *G. asiaticum* proteins into 7459 and 6825 orthologue groups, and large numbers of proteins were not assigned to a group (Additional file 5). Interestingly, the three orthologue families annotated with the highest frequencies were OG5_127031, OG5_126590, and OG5_126561 for *G. yamadae* and OG5_127031, OG5_126588 and OG5_126590 for *G. asiaticum*, indicating that the groups named OG5_127031 and OG5_126590 are well conserved in both species. In the OrthoMCL database, most genes involved in these two groups have the function of transposases and DNA polymerases, respectively, whereas the genes of family OG5_126561 function as ABC transporters, and the genes of OG5_126588 mostly belong to the heat-shock protein family.

### *G. yamadae* and *G. asiaticum* secretome and candidate effectors

**Fig. 10:**
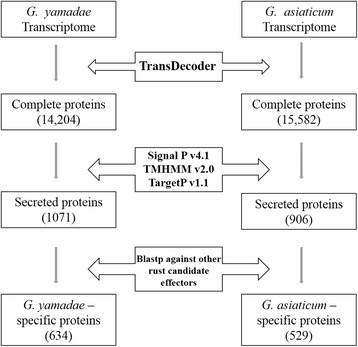
Workflow of secretome prediction using bioinformatics tools for the comprehensive characterization of secreted proteins

Effectors are considered to exhibit the essential ability to manipulate host cell processes and facilitate infection during plant-pathogen interactions. In the present study, we analysed the proteomes of *G. yamadae* and *G. asiaticum* based on 35,102 and 36,343 unigenes using TransDecoder 3.0.1 and predicted 14,204 and 15,582 potential proteins, respectively (Fig. 10). Because effectors are secreted, this first step aimed at identifying candidate effectors was performed to identify secreted proteins. The essential criteria used to identify secreted proteins were the presence of signal peptides (SP), a lack of transmembrane domains and the absence of mitochondria and endoplasmic reticulum-targeting motifs in such proteins [34]. Based on these characteristics, a total of 1071 and 906 secreted proteins were confirmed for *G. yamadae* and *G. asiaticum*, respectively. Datasets of 23,516 candidate effectors for four rust fungi, specifically two *Melampsora* species, *M. lini* and *M. larici-populina,* and two *Puccinia* species*, P. graminis f. sp. tritici* and *P. striiformis f. sp. tritici*, have been mined from their proteomes [35]. A BLASTp search against this dataset showed the presence of 634 and 529 *Gymnosporangium*-specific secreted proteins in the *G. yamadae* and *G. asiaticum* secretomes, respectively, because these proteins showed no significant homology to candidate effectors reported for the other four fungi (e values > e ^−5^). The remaining 437 and 377 secreted proteins are conserved across the dataset. We used OrthoMCL [33] to assign the *Gymnosporangium*-specific secretomes of *G. yamadae* and *G. asiaticum* into 211 and 154 protein families, respectively (Additional file 6: Table S1). An additional characteristic of effectors is that they are typically small proteins rich in cysteines; thus, we manually examined the secretomes of both species and found 758 secreted proteins in the *G. yamadae* secretome with a length shorter than 300 amino acids, and 314 (41.42%) of these were rich (≥4) in cysteine residues (involved in folding and stability). The secretome of *G. asiaticum* contained 661 secreted proteins shorter than 300 amino acids, and 267 (40.39%) of these were rich in cysteines (Additional file 6 and Table S2). Among the putative candidate effectors of *G. yamadae* and *G. asiaticum*, we identified 13 putative CAZymes belonging to eight families in *G. yamadae* and 16 putative CAZymes from 11 families in *G. asiaticum* (Additional file 6: Table S3). To obtain further information on the domains of these secreted proteins, a Pfam search was performed, and the results showed that 30.1% of the cysteine-rich, small secreted proteins (SSPs) of *G. yamadae* contained distinct Pfam-A domains, whereas 34.5% of the cysteine-rich SSPs of *G. asiaticum* contained distinct Pfam-A domains. The Pfam domains that were represented in the *G. yamadae* and *G. asiaticum* SSPs included the following: cysteine-rich fungal effector motif (CFEM), cysteine-rich secretory proteins (CAP), trypsin domains and thaumatin, DBPP-1 (lytic transglycosylase), cutinase, peptidases and Cu-oxidase (Additional file 6: Table S4). Among the small secreted proteins of *G. yamadae* and *G. asiaticum*, 38 and 286 proteins, respectively, showed significant homology to sequences in the Pathogen-Host Interaction (PHI) protein database with an e-value of e^−5^, which indicated effects related to unaffected pathogenicity, virulence and pathogenicity, reduced virulence, and loss of pathogenicity, among others (Additional file 6: Table S5). The assignment of gene families was performed through BLASTp searches against OrthoMCL proteins. The results showed that the largest clusters of protein families containing secreted proteins were as follows: receptor (epidermal growth factor) with a receptor L domain, protein kinase (immunoglobulin I-set domain), kinase (p kinase domain), cpxP (LTXXQ motif), outer membrane porin (gram-negative porin), DNA-binding transcription (SmpA/OmlA family), ATP synthase (ATP synthase B/B′ CF) and GTP (ADP-ribosylation factor family). Ultimately, a total of 34 and seven candidate effectors with Pfam domains exhibiting significant homology to PHI proteins were identified in the *G. yamadae* and *G. asiaticum* secretomes, respectively (Additional file 6: Table S6).

### Orthologues, substitution rates, and transcriptome divergence

The classification of orthologous genes among species is the basis of comparing the differences of species at the transcriptional level. To build a set of orthologues and gene model predictions, OrthoMCL software [33] was used to search and analyse orthologues for their full-length CDSs and was used to screen out one-to-one orthologues for subsequent analysis. A total of 4947 paired orthologues were identified between the two transcriptomes subjected to BLAST analysis using the *Pst* and *Mlp* genome sequences [8, 36], which were the closest phylogenetically published genomes to *G. yamadae* and *G. asiaticum*, with 2302 orthologues (see Additional file 7).

The Ka/Ks ratios of non-synonymous and synonymous substitutions can be used to determine whether a selective pressure exists in a coding gene. A gene with Ka/Ks values less than or more than 1 indicates that the gene in question is subject to purifying or positive selection, respectively [37]. The Ka and Ks values for all 4947 orthologous unigenes in *G. yamadae* and *G. asiaticum* were estimated using the paml-codeml algorithm, and the Ka/Ks ratios were calculated. The mean values of Ka and Ks and the ratios of all orthologous pairs were 0.132, 0.113, and 0.558, respectively. Among the 4947 orthologues, 116 pairs with Ka/Ks values greater than 1 were identified to be under positive selection and are thus regarded as divergent orthologous genes. In addition, 170 pairs with Ka/Ks values of 1 are under neutral selection, and the remaining 4661 genes are subjected to purifying selection (Additional file 8).

**Fig. 11:**
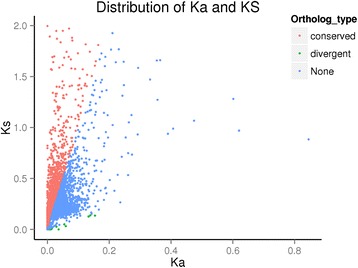
Scatter diagram of Ka and Ks values

Orthologue pairs with Ks values less than 0.1 were further analysed because they been used as a benchmark to avoid the inclusion of paralogs [38]. Hence, taking a more accurate threshold of Ka/Ks < 0.1 [39], the eligible orthologues were considered conserved orthologous genes (Fig. 11 and Additional file 8)

Evolutionary distances are expressed in terms of the number of base substitutions per site. For coding regions, substitutions might be further classified as occurring at synonymous (silent) and non-synonymous sites, and the corresponding distances are referred to as Ks and Ka, respectively. Distances, K, might be converted to rates, r, using the equation r = K/(2 T), where T is the divergence time between the two species [40]. The simple formula T = K/2r [41] was used to roughly estimate the divergence time (T) between *G. yamadae* and *G. asiaticum*. The average synonymous substitution r estimated with Langley Fitch (LF) algorithms and a Eurotiomycetes and Sordariomycetes (ES) calibration point of 400 Myr was 28.5 ± 28.7 × 10^−9^ in Eurotiomycetes fungi, a monophyletic class of Ascomycota [42]. Although there is deviation between Ascomycota and Basidiomycota, the scope is between 10^−9^ and 10^−8^, which can be used to roughly estimate the synonymous mutation rate in *Gymnosporangium* species. K is the genetic divergence expressed in terms of the mean number of synonymous substitutions between orthologues [19]. In our case, the evolutionary tree model was constructed using 23 types of maximum likelihood methods to calculate the Akaike Information Criterion (AIC) and the (Bayesian Information Criterion (BIC) values; minimum values were selected to construct the phylogenetic tree and K value, and the K value between *G. yamadae* and *G. asiaticum* rounded to 0.15 (Additional file 9). Based on the above results, we calculated that the age of the speciation event between both species was approximately 5.2 Mya.

### Important gene families and metabolic pathways among orthologues of *G. yamadae* and *G. asiaticum* under adaptive evolution

A GO enrichment analysis (abbreviated GO Seq [43]) was performed to analyse divergent (Ka/Ks > 1) and conserved (Ka/Ks < 0.1) genes in order to identify rapidly evolving and conserved genes involved in biological processes, molecular functions, and cellular components. In this study, 64 GO terms assigned to 21 pairs of orthologues were overrepresented (*P value* < 0.05) under positive selection. These results suggest that among the candidate genes under positive selection, approximately one-third share high sequence similarity with homologs annotated to cell division and the reproduction process. A total of 61 GO terms were overrepresented (*P value* < 0.05) under purifying selection. Biological process (GO: 0008150), cellular process (GO: 0009987) and metabolic process (GO: 0008152) were the three top GO terms (Additional file 10). Using the hypergeometric test, all significant enrichment pathways for divergent and conserved homologous genes with respect to all annotated genes were analysed. In our case, 34 orthologous genes (positive selection) showed significant associations with 19 pathways (corrected *P value* < 0.05; Additional file 10). The three most enriched pathways were peroxisomes (ko04146), ribosomes (ko03010), and nitrogen metabolism (ko00910). A total of 547 pairs of orthologues (purifying selection) were enriched in 20 pathways. The three most enriched pathways were the ribosome (ko03010), spliceosome (ko03040) and proteasome (ko03050) pathways.

## Discussion

### RNA-Seq analysis of the *G. yamadae* and *G. asiaticum* transcriptomes

In this study, we generated two genome-wide transcriptomes during the telial life stage of *G. yamadae* and *G. asiaticum*. Recent rust RNA-Seq-based transcriptomic analyses have focused on comparing different infection stages in the telial hosts of several rust fungi, such as *M. larici-populina*, *P. graminis* f. sp. *tritici*, *P. striiformis* f. sp. *tritici*, *H. vastatrix*, *P. pachyrhizi* and *U. appendiculatus*, and considerable progress has been made in understanding the patterns of gene expression during the infection process [9, 12, 13, 15, 16]. Additionally, a comprehensive RNA-Seq analysis of *C. ribicola* with its host plant, *Pinus monticola*, which was reported in 2015, generated a genome-wide transcriptome and secretome of *C. ribicola* at three life stages [10].

The present study constitutes the first comparative analysis of the transcriptomes of two related *Gymnosporangium* species, for which there are no genomic resources. In our case, we used a consensus assembly approach for three biological replicates of each species to eliminate errors within the group and to enhance the reliability of the transcripts. A large proportion of the reads were mapped to the reference transcriptomes (83.98 and 78.32%), which demonstrates the high quality of the Trinity assembly and indicates that the data produced via our processing approach using telial spore samples were satisfactory for non-model organisms without a reference genome sequence.

Our statistical analysis revealed that 18,467 putative proteins with an average length of 233 bp and 15,037 opening reading frames (ORFs) were complete proteins (47.93%) in *G. yamadae*, and 17,769 putative proteins with an average length of 241 bp and 15,248 ORFs were complete (60%) in *G. asiaticum*. The proportions of complete proteins and truncated proteins were similar to those provided in a recent study on *C. ribicola* [10]. Furthermore, approximately half of the hitless unigenes were short sequences between 200 and 500 bp in length, as was expected for the overrepresented set described in Fig. 3. Some researchers have recently indicated that some non-hit unigenes are noncoding RNAs, 5′ or 3′ UTRs, or intron sequences [44, 45].

### *G. yamadae* And *G. asiaticum* candidate effectors

Rust fungi are obligate biotrophs that derive their nutritional resources entirely from living host plant cells. Similar to other plant-associated pathogens, rust fungi secrete effector proteins via specialized feeding structures known as haustoria during infection [46]. The haustorium–host cell interface appears to mediate a dynamic interaction involving extensive trafficking of nutrients as well as signalling and defence molecules [47]. The development of a method to isolate haustoria from infected tissues was a remarkable breakthrough in the attempts to understand haustorial function and identify haustoria-specific genes [48]. Only six effector proteins have been identified to date: AvrM, AvrL567, AvrP123, and AvrP4 in the flax rust *Melampsora lini*, RPT1 in the bean rust *Uromyces fabae*, and PGTAUSPE-10-1 in the wheat stem rust fungus *Puccinia graminis* f. sp. *tritici* [49–52]. Most fungal effectors exhibit the characteristics of secreted proteins, are rich in cysteine and have lengths shorter than 300 amino acids [53, 54]. Thus far, no effectors have been identified in *Gymnosporangium* species, making it difficult to study the dynamic process of pathogen-host interactions and to achieve a clear understanding of how the effectors function in modulating host defence and metabolism in these species.

The classical methods for identifying fungal effectors, such as map-based cloning, analysis of fungal secretomes during infection, identification of HR-inducing pathogen genes, and mutagenesis and screening of expressed sequence tag (EST) libraries [53], are labour intensive. Thus, computer-based pipelines have been developed to identify candidate effectors based on certain characteristics, such as small size, the presence of signal peptides, and being rich in cysteine. With this criteria, 1071 (7.5%) and 906 (5.8%) secreted proteins were identified in *G. yamadae* and *G. asiaticum*, respectively, and these numbers and proportions of secreted proteins are similar to those provided in a former report on the secretome predictions of four rust fungi, including *M. lini*, *M. larici-populina*, *P. graminis* f. sp. *tritici*, and *P. striiformis* f. sp. *tritici* [35]. Among the secreted proteins identified in this study, 314 and 267 were cysteine-rich, small secreted proteins in *G. yamadae* and *G. asiaticum*, respectively*.* The CAZymes involved in the decomposition of plant cell wall components are essential for the success of colonization and acquisition of nutrients from the host, and their content reflects the lifestyle of this fungi. According to the annotation in the dbCAN database, the CAZyme-like proteins were mostly assigned to the glycosyl hydrolase families, similar to findings in other biotrophic fungi [10, 54]. Through further searches using the Pfam database, a large number of the domains of these SSPs were found to be conserved in both *G. yamadae* and *G. asiaticum*, such as the CAP, DBPP-1, cutinase, peptidase, Cu-oxidase, trypsin and thaumatin domains, and several fungal proteins have been reported to be functionally involved in fungal pathogenicity [10, 13, 52]. The BLAST results for the PHI dataset combined with the Pfam search results allowed the final 34 and seven candidate effectors to be identified from the transcriptomes of *G. yamadae* and *G. asiaticum*, respectively. Of the 34 candidate effectors in *G. yamadae*, ten candidate effectors belonged to eight families that have been identified as *Gymnosporangium*-specific proteins. These ten families have not been found in any other rust fungi [8, 10, 35], which showed that these families are likely *Gymnosporangium*-specific. Of the seven candidate effectors in *G. asiaticum*, one protein (Gene. 15,136__GYA_c13708_g1__g.15136__m.15136) has been identified as genus-specific compared with the *Melampsora* and *Puccinia* genera, and this protein belongs to glycoside hydrolase family 19 (GH19). However, the most abundant families of CAZyme-like proteins found in other rust effectors were GH5, GH18 and GH47 [8, 10, 17]. The GH19 family appears to be a *Gymnosporangium*-specific family, and the candidate effector is novel. Due to incomplete sequences and missed signal peptides, the number of candidate effectors identified in our study was markedly lower than expected compared with other recent research [8, 10, 55]. As the dataset of the determinants involved in plant-pathogen interactions expands, many more candidate effectors will be mined from the rust fungi genomic sequences.

### Estimation of the divergence of *G. yamadae* and *G. asiaticum*

As more genomic and transcriptome information becomes available, the divergence time between two related species at the molecular level has been frequently estimated through the calculation of synonymous (Ks) substitutions following a simple formula: T = K/2r [41]. This estimation method is widely used in plants and animals [19, 23]; however, few reports have involved the time of divergence based on two related fungi, particularly rust fungi. It is generally recognized that estimates of the time when two organisms last shared a common ancestor are based on suppositions arising from fossil records [56]. Nevertheless, considering a situation where there is no adequate fossil dating of the divergence of two species, estimating the divergence time based on the coding region of orthologous genes can still be regarded as an effective method. According to the fossil calibration of the genus *Ravenelia* (55.8–40.4 Mya, fossil record), the divergence of *Gymnosporangium* species from the genus *Ravenelia* of Basidiomycetes occurred 51.7–44.3 Mya during the Eocene epoch of the Palaeogene period in the Cenozoic era, and *G. yamadae* proved to be the most recently divergent species [57]. This study constitutes the first use of the peak synonymous rates (Ks) for orthologous transcript pairs for estimating the time of divergence between species of rust fungi. The divergence time estimated for *G. yamadae* and *G. asiaticum* fell within the range of 51.7 to 44.3 Mya, which indicates the effectiveness of calculating the divergence time of *Gymnosporangium* species using the equation r = K/(2 T). Estimates for the divergence age of rust fungi have varied considerably. A study on the time tree of life indicated that families, genera and species in the Basidiomycotina diverged *c.* 111, 98 and 6 Ma, respectively [58]. This is not consistent with a later research, in which the authors estimated the divergence time of more than 20 genera of rust fungi by using a molecular clock, calibrated against estimated divergence time for the hosts of rust fungi, showing that families, genera and species of rust fungi within two suborders Uredinineae sensu Aime and Melampsorineae sensu Aime [59] diverged *c.* 38–46, 22–37 and 0.3–17 Ma, respectively, the divergence time interval of species in this study validated the reliability of the result in our study. This divergence time is a rough estimate based on the coding region of orthologous genes. Nevertheless, this estimation is also effective because fossils are not available to accurately determine the divergence time of these two *Gymnosporangium* species. Moreover, this estimation serves as a basis for future studies of genetic divergence between these two species.

### Gene families and pathways associated with the telial stage of *G. yamadae* and *G. asiaticum*

The telial stage of rust fungi is crucial in its lifecycle because karyogamy and meiosis occur in this stage. Karyogamy and meiosis are essential cellular processes and play a fundamental role in generating genetic diversity by promoting recombination between chromosome homologues [60]. In haploid plant pathogen fungi, meiosis drives extraordinary genome plasticity and facilitates rapid adaptation to changing environments [61, 62]. To gain further insight into the physiological and molecular factors playing a role in the telial stage, we focused on several gene families shared between *G. yamadae* and *G. asiaticum* based on functional annotations.

#### Stress-related genes

In late winter, teliospores of *G. yamadae* and *G. asiaticum* extrude overwintered structures, such as branch galls and stem swelling or needles lesions, respectively. A functional KOG analysis of *G. yamadae* and *G. asiaticum* genes indicated that many transcripts are involved in the adaptation to adverse conditions due to the low temperatures that the teliospores have to tolerate. Several genes potentially related to cold-temperature tolerance were identified, including 26 and 38 aquaporins, respectively, in *G. yamadae* and *G. asiaticum* (Additional file 5). Aquaporins have been shown to function in desiccation and freezing tolerance in microorganisms, including bacteria, yeast, and fungi [63]. Interestingly, a previous transcriptome analysis of poplar rust *M. larici-populina* in the telial stage also revealed that many transcripts encoding aquaporins are specifically expressed in telia, suggesting that telia support survival of the spores and prevent osmotic damage of the cells due to freezing [58]. We also found six and one calcium-transporting ATPases, respectively, in *G. yamadae* and *G. asiaticum* (Additional file 5). Calcium-transporting ATPases comprise a transporter family that serve to transport Ca^2+^ from the cell, which is related to the *M. larici-populina* overwintering process [60]. Furthermore, we also found several genes in both *G. yamadae* and *G. asiaticum* encoding multicopper oxidase laccase-like proteins, which are supposedly involved in the biosynthesis of melanin pigment. The multicopper oxidase laccase-like proteins were also identified in an EST library of *P. triticina* and *M. larici-populina* teliospores [60, 64]. Teliospores are highly melanized structures, and melanin is thought to provide protection against adverse environmental conditions [60].

#### Genes related to meiosis

Meiosis is a crucial cellular process that occurs in teliospores. As a specialized type of cell division, it functions as an evolutionary adaptation for DNA repair. In addition, it is also a vital process during which genetic diversity is generated by promoting recombination between chromosome homologues. In fungi, meiosis can drive genome plasticity and facilitate rapid adaptation to changing environments [60]. Several genes have been reported to be conserved eukaryotic meiotic genes, including *Rec8*, *Mre11*, *Rad50*, *Rad51*, *MutS4*, *MutS5*, *Spo11*, *Mnd1*, and *Mlh1* [65]. In this study, we identified several transcripts predominantly involved in the meiosis process, including DNA repair proteins Rad50 and, Rad51, mismatch repair ATPase family protein (MutS), and a DNA mismatch repair protein of the MLH1 family, in both *G. yamadae* and *G. asiaticum* (Additional file 5). Similar to our findings, four telia-specific karyogamy and meiosis-related genes with higher transcript levels were detected in the teliospores of *M. larici-populina* [60].

#### Cell wall modification enzyme families

Fungal cell walls are complex polysaccharide structures composed mainly of chitin, glucans and other major components [66]. GHs, PLs, and CEs are enzymes involved in the formation, remodelling or degradation of cell walls and play a fundamental role in plant-fungal pathogen systems [67]. Nine GH families (GH2, 13, 18, 20, 30, 33, 73, 84 and 101) have been demonstrated to act as virulence factors in pathogens [68]. Moreover, at least 44 GH families have been reported in fungi [69]. However, biotrophic fungi, such as rust fungi, possess a reduced number of GH enzymes, consistent with the necessity of minimizing host cell wall damage to avoid triggering a plant response during colonization [8]. In this study, we identified a total of 147 and 150 transcripts in *G. yamadae* and *G. asiaticum*, respectively, with similarity to the GH superfamily, and these belong to 42 and 43 families, respectively (Additional file 5). The most abundant families in both species were GH5 (cellulases/hemicellulase), GH16 (β-1,3-glucanas), GH47 (α-1,2-mannosidases), and GH18 (chitinases), which is consistent with recent results obtained in *Mlp*, *Pgt*, *Pst* and *C. ribicola* [8, 10, 17]. The high enrichment of these enzyme families suggests a potential role in the degradation and loosening of plant cell walls. We also found several conserved orthologues (OG09504, OG09756 and OG09106; under purifying selection) between *G. yamadae* and *G. asiaticum* that contribute to eight GO terms associated with functions and processes related to chitin catabolism (Additional file 10). Thus, rust fungi appear to have evolved a stable strategy involving the modification of their own cell wall to impede identification by host plant cells, which would trigger an immune response.

#### Carbohydrate metabolism

Genes involved in the carbohydrate metabolism process were abundant in both *G. yamadae* and *G. asiaticum* (Fig. 9). The transcripts classified in this category were mostly enriched in glycolysis/gluconeogenesis, the citric acid cycle (TCA cycle) and the pyruvate metabolism pathway (Additional file 5). Recent research has indicated that transcripts involved in the primary pathway of energy production comprising glycolysis, TCA cycle, and oxidative phosphorylation are mostly upregulated in haustoria [17], and similar observations were made in the obligate biotrophs *U. fabae* and *Blumeria graminis*, in which genes related to glycolysis are upregulated during the parasitic stage [70, 71]. Furthermore, a four-time-point (pre-penetration, colonization, and sporulation stages) transcriptional analysis conducted in *P. pachyrhizi* indicated that genes encoding enzymes involved in energy production and carbohydrate metabolism were abundant at all-time points [12]. The genes in metabolism process were overrepresented in the telial stage of *G. yamadae* and *G. asiaticum*, corresponding to the results found in haustoria and other stages. This could be a consequence of the nutritional status of telial stage when the energy is greatly demanded to overcome the harsh situation that the teliospores have to tolerate.

#### Peroxisome pathway

Peroxisomes are essential organelles found in all eukaryotic cells that play a key role in redox signalling and lipid homeostasis and contribute to many crucial metabolism processes such as the breakdown of very long chain fatty acids through beta-oxidation [72–77]. The final product of fatty acid oxidation is acetyl-CoA, which can directly enter the glyoxylate cycle, where lipids are converted into carbohydrates through the action of several enzymes. Five orthologous genes under purifying selection were associated with this pathway, and the most important of these was AGXT, which is involved in carbohydrate, energy, and amino acid metabolism related to alanine-glyoxylate transaminase. These results suggest that the top enriched pathway plays a significant role in maintaining lipid homeostasis through several redox reactions. With respect to carbohydrates, gene families encoding major facilitator proteins are reduced in rust fungi, whereas some of these genes are upregulated during leaf colonization for the acquisition of sugar from leaves [8]. Thus, we can speculate that AGXT is overrepresented during the teliospore stage of *Gymnosporangium* species to obtain energy to germinate for the formation of basidiospores.

### Shortcomings of the current study and future directions

As previously mentioned, the 101-Mb genome of *M. larici-populina* and the 89-Mb draft genome of *P. graminis* f. sp. *tritici* were first sequenced by the Joint Genome Institute and the Broad Institute, respectively [8]. Since then, with the sequencing and publication of draft genomes of other rust fungi, more genomic resources are becoming available. Nevertheless, the genome sequence progresses for a large proportion of rust fungi were impeded due to an unclear information about their genome sizes. Flow cytometry was recently applied to estimate the genome sizes of several rust fungi, and at that time, *G. confusum* had the largest fungal genome yet reported, with a size of 893.2 Mbp [7]; however, this result was eclipsed by a subsequent estimation of the genome size of *Uromyces bidentis* in 2015, yielding the largest rust fungal genome to be reported [78]. The draft genome size estimation of *G. confusum* provided only limited information for the genus *Gymnosporangium*, and notable information remains to be gleaned.

This transcriptome study constitutes a first attempt to gain insights regarding the biology of these very important fungal pathogens, and resolving their genome sequences would be the next step toward completing our knowledge about some genus-specific questions. For example, by comparing the differences in the genome sequences of these fungi, we could speculate why the species of this genus possess a unique host type in their lifecycles. As mentioned previously, this genus differs from other rust fungi in that its telial host is a gymnosperm, whereas the spermogonia and aecial hosts are dicotyledonous plants. Furthermore, *Gymnosporangium* species exhibit another difference from other macrocyclic rusts: most species of *Gymnosporangium* lack urediospores. Explaining these biological differences with the support of genomic data would be ideal. Hopefully, with the development of novel sequencing technologies and the declining costs of next-generation sequencing, increasing genomic resources and transcriptome sequences for this genus will become available in the near future, and these questions will be better resolved.

## Conclusion

In this study, a de novo assembly and comparative analysis of the transcriptomes of the two rust species *G. yamadae* and *G. asiaticum* were conducted using RNA-seq. We identified several orthologous genes, and upon annotation of these two species, we produced a broad overview of the expressed genes. Calculation of Ka/Ks ratios allowed identification of the orthologous genes under positive, neutral and purifying selection. An evolutionary analysis of these two species provided a relatively precise divergence time despite the absence of a fossil record. Moreover, this study identified a repertoire of candidate effectors and other pathogenicity determinants.

Due to the lack of a reference genome sequence for *Gymnosporangium*, the available gene function database information is fairly scarce. A high proportion of orthologous genes have been identified in the two *Gymnosporangium* species and in the rust pathogens *P. graminis* f. sp. *tritici* and *M. larici-populina*, and these genes appear to be expressed as hypothetical proteins. Further study is needed for the in-depth exploration of their latent involvement in pathogenicity. Furthermore, future studies should focus on identifying the molecular mechanisms governing the interactions between *Gymnosporangium* species and their hosts, particularly on the substantial transcriptome changes in both organisms. Finally, the characterization of essential molecular factors involved in disease development will provide useful management tools for the prevention and control of the economically significant rust diseases caused by these *Gymnosporangium* species.

## Methods

### Rust sample collection

Teliospores of *G. yamadae* and *G. asiaticum* were collected from an infected cypress tree (*Juniperus chinensis* L.) prior to telial horn gelatinization at Northwest A&F University, Yangling, Shaanxi, China, in March 2016. Each sample was harvested with three biological repeats, frozen in liquid nitrogen, and stored at −80 °C prior to RNA extraction. Each biological repeat was used for RNA extraction and RNA-Seq library construction.

### RNA-Seq library preparation and sequencing

An approximately 50-mg sample of teliospores from each replicate was used for total RNA extraction following a previously described protocol [10]. The total RNA was treated with a DNA-free™ DNA Removal Kit (Ambion, AM1906) (Ambion, AM1906) to remove contaminating genomic DNA. The resulting RNA pellets were suspended in approximately 30 μl of DEPC-treated water. RNA degradation and contamination were monitored in 1% agarose gels. RNA purity was checked using a Nano-Photometer spectrophotometer (IMPLEN, CA, USA). Prior to cDNA library synthesis, the RNA concentration was measured using a Qubit® RNA Assay Kit with a Qubit® 2.0 Fluorometer (Life Technologies, CA, USA), and RNA integrity was assessed using an RNA Nano 6000 Assay Kit with an Agilent Bioanalyser 2100 system (Agilent Technologies, CA, USA). The RNA integrity numbers (RINs) of each sample were as follows: 8.1, 7, and 8.6 for the three biological repeats of *G. yamadae* and 8.8, 8 and 8.7 for the three biological repeats of *G. asiaticum*, respectively. Hence, all RNA samples could be used for RNA-Seq platform analysis.

A total of 3 μg of RNA per sample was used as input material for RNA sample preparation. Six sequencing libraries from the three replicates of *G. yamadae* and *G. asiaticum* were generated using a NEBNext® Ultra™ RNA Library Prep Kit for Illumina (NEB, USA) following the manufacturer’s recommendations. Clustering of the index-coded samples was performed with the cBot Cluster Generation System using a TruSeq PE Cluster Kit v3-cBot-HS (Illumina) according to the manufacturer’s instructions. After cluster generation, the library preparations were sequenced with an Illumina HiSeq 2000 platform by Novogene Bioinformatics Technology Co., Ltd. (Beijing, China), and 100-bp paired-end reads were generated.

### Reprocessing of Illumina raw data and de novo transcriptome assembly

The raw sequenced reads were processed using Trimmomatic software [79]. In this step, clean reads were obtained by removing reads containing adapters, poly-Ns (≥10%) and low-quality reads (sQ ≤ 5) from the raw data. Simultaneously, the Q20, Q30, GC content and sequence duplication level of the clean data were calculated. All downstream analyses used clean data with high quality.

The resultant filtered and trimmed sets of high-quality reads for the three libraries of each species were combined for de novo assembly using the Trinity software package [18], with min_kmer_cov set to 2 by default and all other parameters set to the default values. Trinity combined reads with certain lengths of overlap to form longer fragments without Ns, which were called contigs. Given the algorithm and the principle of Trinity, the various transcripts of the same gene may originate from splice variants (isoforms), alleles, the same gene copy, homologue, orthologue, etc. After the elimination of redundancy, these contigs were subjected to further sequence clustering to form longer sequences without Ns. Such sequences were defined as unigenes [80]. To calculate an abundance estimate of each unigene, clean data were mapped back onto the assembled transcriptome using RSEM software [22], and the read counts for each unigene were obtained from the mapping results and adjusted to the FPKM to facilitate the comparison of transcript levels assigned to each gene between samples. The FPKM value of each unigene of the three biological samples were normalized by taking the average value. Unigenes with FPKM values greater than 0.3 are likely to be expressed. To avoid false-positive estimation of gene expression, unigenes with a FPKM value of at least 1 were retained for downstream analysis.

Meantime, quantitative saturation curves were used to confirm the amount of sequencing data needed for these two species. Taking the gene expression level of 100% mapped fragments as the final standard, we conducted quantitative analysis for 10%, 20%, 30%…90% mapped fragments respectively. Compare the single gene FPKM value calculated under the condition of each percentage (10%, 20%, 30%…90%) to the final gene expression level of corresponding genes, if the difference is less than 10%, the gene under this condition is regarded as being accurately quantified (Additional file 11). In these figures, the abscissa represents the ratio of reads on genome to total mapped fragment, the ordinate represents the genes quantitative error within 10% accounted for the proportion of the total number of genes, and lines with different colors represent different FPKM intervals. The results showed that when the sequencing data were approaching 6G, the curve was reaching saturation, which indicated that our data covered the large feasible portion of genes and met the requirements for analysis.

### Prediction of secreted proteins

Putative ORFs were identified within transcripts using TransDecoder, with a minimum protein length of 50. Complete ORFs were scanned for signal peptides using SignalP v4.1 [81] with default parameters. The resulting peptides were then scanned for transmembrane helices and mitochondria-targeted sequences using the TMHMM v2.0 programme [82] and TargetP v1.1 [83], respectively. Reciprocal BLASTp analysis was performed against a dataset of other candidate effectors [8, 35, 36, 84]. Putative CAZymes were identified through BLAST searches against the CAZy database using the dbCAN v3.0 HMM-based CAZy annotation server [32]. The assignment of gene families was performed with BLASTp against OrthoMCL proteins with a cut-off of e^−5^ [33]. The Pfam search was performed using the online service of the European Bioinformatics Institute [EMBL-EBI] at the gathering threshold with a cut-off of e^−3^ and dom of e^−3^.

### Gene functional annotation

The assembled unigenes of *G. yamadae* and *G. asiaticum* were annotated via mapping to several public databases. To describe the predicted genes of assembled unigenes, they were aligned against and compared with the Nr, Nt, KOG and KEGG orthologue (KO) databases using BLASTX with a significance threshold of e ≤ 10^−5^. The unigenes were also queried against the Pfam database using default parameters. The GO terms describing biological processes, molecular functions, and cellular components for functional categorization were analysed using Blast2 go software [27]. The E-value filter for GO annotation was e^−6^. Pathway assignments were performed by sequence searches against the KO database and by using the BLASTX algorithm with a threshold of e^−5^. After these processes, proper GO terms and KO pathways were generated.

### Identification of orthologous gene groups and calculation of ka/Ks ratios

The CDSs for each putative unigene were extracted based on the BLASTX results. ESTScan software was then used to determine the direction of sequences that did not yield alignment results, and the resultant CDSs extracted from their respective unigenes were translated into amino acid sequences using the standard codon table. Self-to-self BLASTP was conducted for all amino acid sequences with a cut-off E-value of e^−5^. Orthologous groups were constructed from the BLASTP results using OrthoMCL v2.0.3 software [33] with default settings. In genetics, the Ka/Ks ratio is the ratio of the number of non-synonymous substitutions per non-synonymous site (Ka) to the number of synonymous substitutions per synonymous site (Ks), which can be used as an indicator of the selective pressure acting on a protein-coding gene. Comparisons of homologous genes with high Ka/Ks ratios are considered to be evolving under positive selection, whereas homologous genes with Ka/Ks ratios close to 0 contain mostly synonymous substitutions, which indicates that these genes are under heavy selective pressure, likely due to a conserved function. Ka/Ks calculations were performed using PAML [85] software with default settings.

### GO and KEGG enrichment analysis

GO enrichment analysis of the groups of divergent and conserved gene orthologues was implemented using the GOseq R package-based Wallenius non-central hypergeometric distribution [86], which could adjust for gene length bias in the divergent and conserved gene orthologue groups. KEGG [31] is a database resource for understanding high-level functions and utilities of biological systems, such as cells, organisms and ecosystems, based on molecular-level information, particularly for large-scale molecular datasets generated via genome sequencing and other high-throughput experimental technologies. We employed KOBAS [43] software to test the statistical enrichment of the divergent and conserved gene orthologue groups in KEGG pathways.

## Additional files

Additional file 1: The statistics of gene expression level of each sample of *G. yamadae* and G. asiaticum. This file provides the read count and corresponding FPKM values of each genes respectively in *G. yamadae* and G. asiaticum when mapped to the reference transcriptome. (ZIP 5443 kb)

Additional file 2: Assembled *G. yamadae* and G. asiaticum transcripts. This file provides the fasta sequences of the 49,318 *G. yamadae* and 54,742 G. asiaticum transcripts. (ZIP 11573 kb)

Additional file 3: Unigenes of *G. yamadae* and G. asiaticum. This file provides the list of 35,102 *G. yamadae* and 36,343 G. asiaticum unigenes. (ZIP 15529 kb)

Additional file 4: CDS prediction analysis. This file provides the predicted CDSs and amino acid sequences based on annotation in a protein database or using ESTScan software. (ZIP 47387 kb)

Additional file 5: Unigene functional annotation of *G. yamadae* and G. asiaticum. This file provides the unigene annotations based on seven databases: Nr, Nt, Pfam, KOG/COG, SwissProt, GO and KEGG. (ZIP 77245 kb)

Additional file 6: Candidate effectors. This file provides the small secreted proteins and candidate effectors predicted in *G. yamadae* and G. asiaticum. (ZIP 184 kb)

Additional file 7: Analysis of orthologous genes. This file provides the orthologous genes identified by OrthoMCL as well as selected one-to-one orthologous genes. (ZIP 312 kb)

Additional file 8: Ka/Ks analysis. This file provides the conserved and divergent orthologous genes based on the calculation of Ka/Ks values for each orthologous gene. (ZIP 33 kb)

Additional file 9: Divergence time estimation for the two Gymnosporangium species. This file provides the AIC, AICc and BIC values calculated by 23 computational models as well as the evolutionary tree constructed with the minimum model. (ZIP 2 kb)

Additional file 10: GO and KEGG pathway enrichment analysis. This file provides the GO enrichment and KEGG pathway enrichment of conserved and divergent orthologous genes. (ZIP 2456 kb)

Additional file 11: This file provides the saturation curves of the gene expression levels of *G. yamadae* and G. asiaticum. (ZIP 2739 kb)

## Abbreviations

AIC: Akaike information criterion
BIC: Bayesian information criterion
BLAST: Basic local alignment search tool
CAP: Cysteine-rich secretory proteins
CAZymes: Carbohydrate-active enzymes
CBMs: Carbohydrate binding modules
CDS: The coding sequences
CEs: Carbohydrate esterases
CFEM: Cysteine-rich fungal effector motif
EST: Expressed sequence tag
FPKM: Fragment per kilobase per transcript per million mapped reads
GHs: Glycosyl hydrolases
GO: Gene ontology
GTs: Glycosyl transferases
KAAS: KEGG automatic annotation server
KEGG: Kyoto encyclopaedia of genes and genomes
KOG: Eukaryotic orthologue
Nr: NCBI non-redundant protein sequence
Nt: NCBI nucleotide sequences database
ORFs: Opening reading frames
Pfam: Protein family
PLs: Polysaccharide lyases
RINs: RNA integrity number
RSEM: RNA-Seq using expectation maximization
