# Supplementary figures and images for: Comparative transcriptome analysis and identification of candidate effectors in two related rust species (Gymnosporangium yamadae and Gymnosporangium asiaticum)
Source: BMC Genomics. 2017 Aug 23;18:651. doi: 10.1186/s12864-017-4059-x (PMC5567642; doi:10.1186/s12864-017-4059-x)

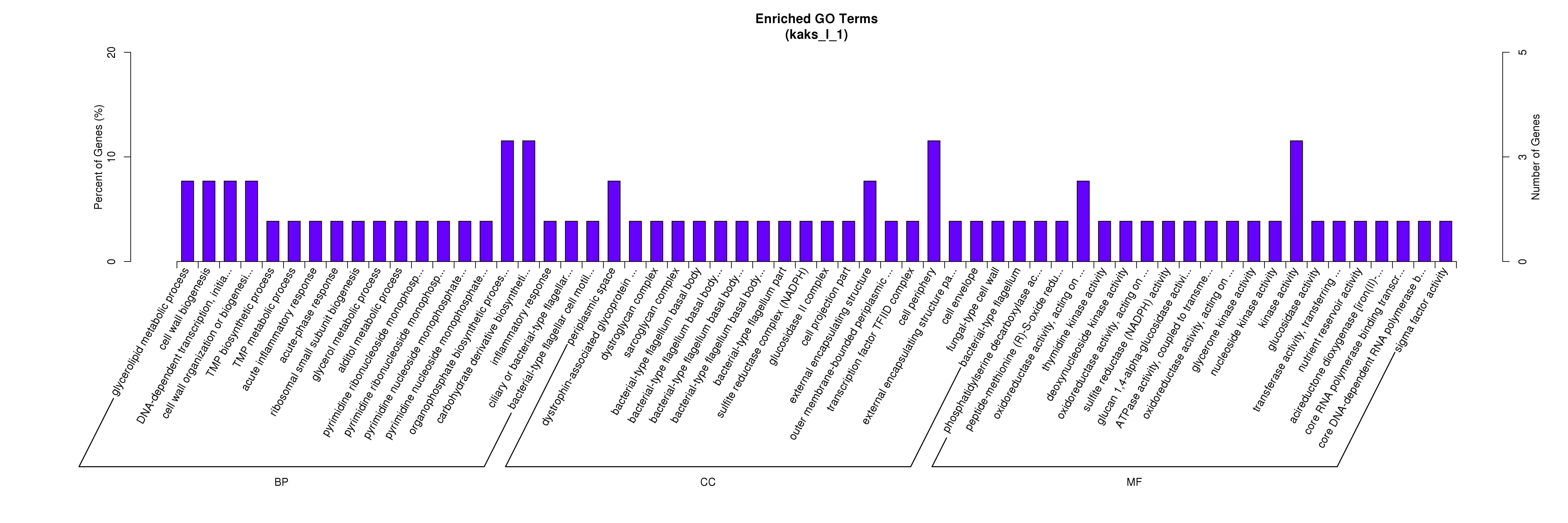

Supplement: Supplementary file 10 — GO and KEGG pathway enrichment analysis. This file provides the GO enrichment and KEGG pathway enrichment of conserved and divergent orthologous genes. (ZIP 2456 kb) [file 12864_2017_4059_MOESM10_ESM.zip › Additional file 10/kaks_l_1.CAD_Enriched_GO_classification.png]

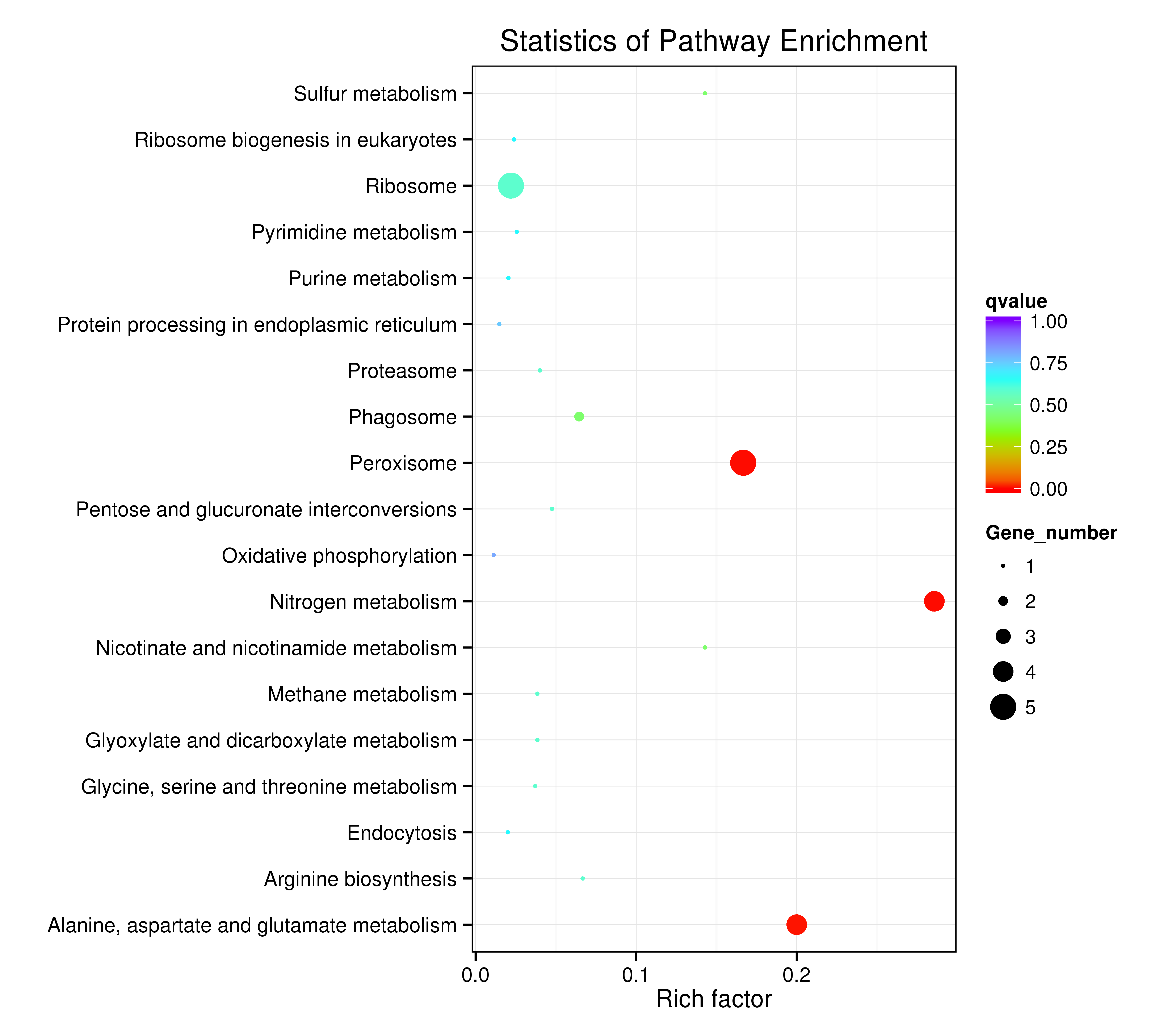

Supplement: Supplementary file 10 — GO and KEGG pathway enrichment analysis. This file provides the GO enrichment and KEGG pathway enrichment of conserved and divergent orthologous genes. (ZIP 2456 kb) [file 12864_2017_4059_MOESM10_ESM.zip › Additional file 10/kaks_l_1.CAD_enriched_KEGG_pathway_scatterplot.png]

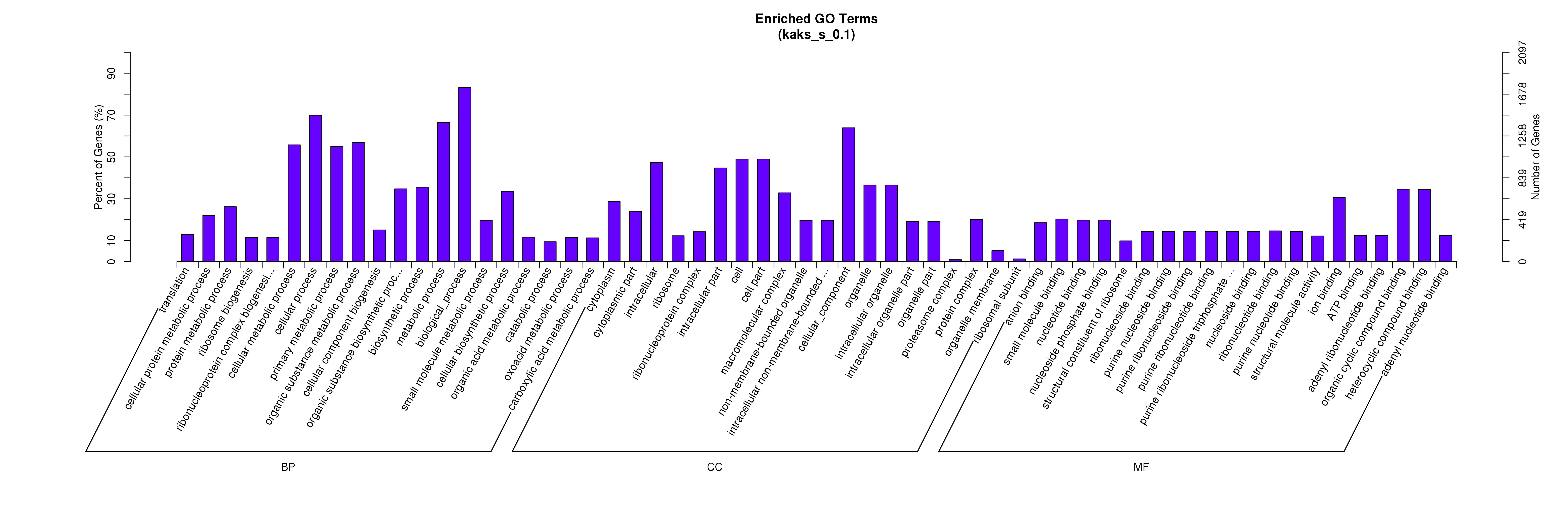

Supplement: Supplementary file 10 — GO and KEGG pathway enrichment analysis. This file provides the GO enrichment and KEGG pathway enrichment of conserved and divergent orthologous genes. (ZIP 2456 kb) [file 12864_2017_4059_MOESM10_ESM.zip › Additional file 10/kaks_s_0.1.CAD_Enriched_GO_classification.png]

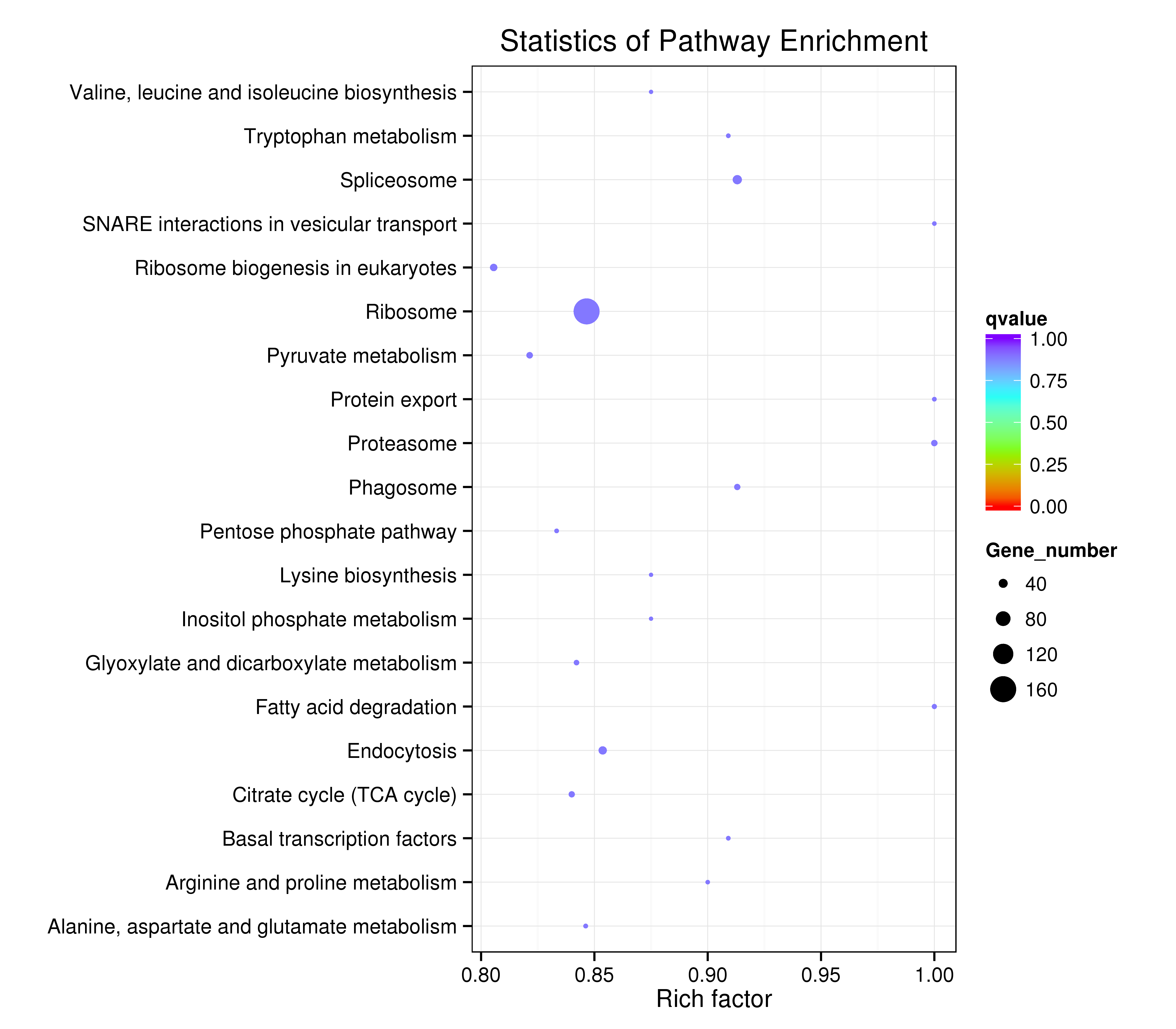

Supplement: Supplementary file 10 — GO and KEGG pathway enrichment analysis. This file provides the GO enrichment and KEGG pathway enrichment of conserved and divergent orthologous genes. (ZIP 2456 kb) [file 12864_2017_4059_MOESM10_ESM.zip › Additional file 10/kaks_s_0.1.DEG_enriched_KEGG_pathway_scatterplot.png]

Saturation Curve (DSXGA\_1)

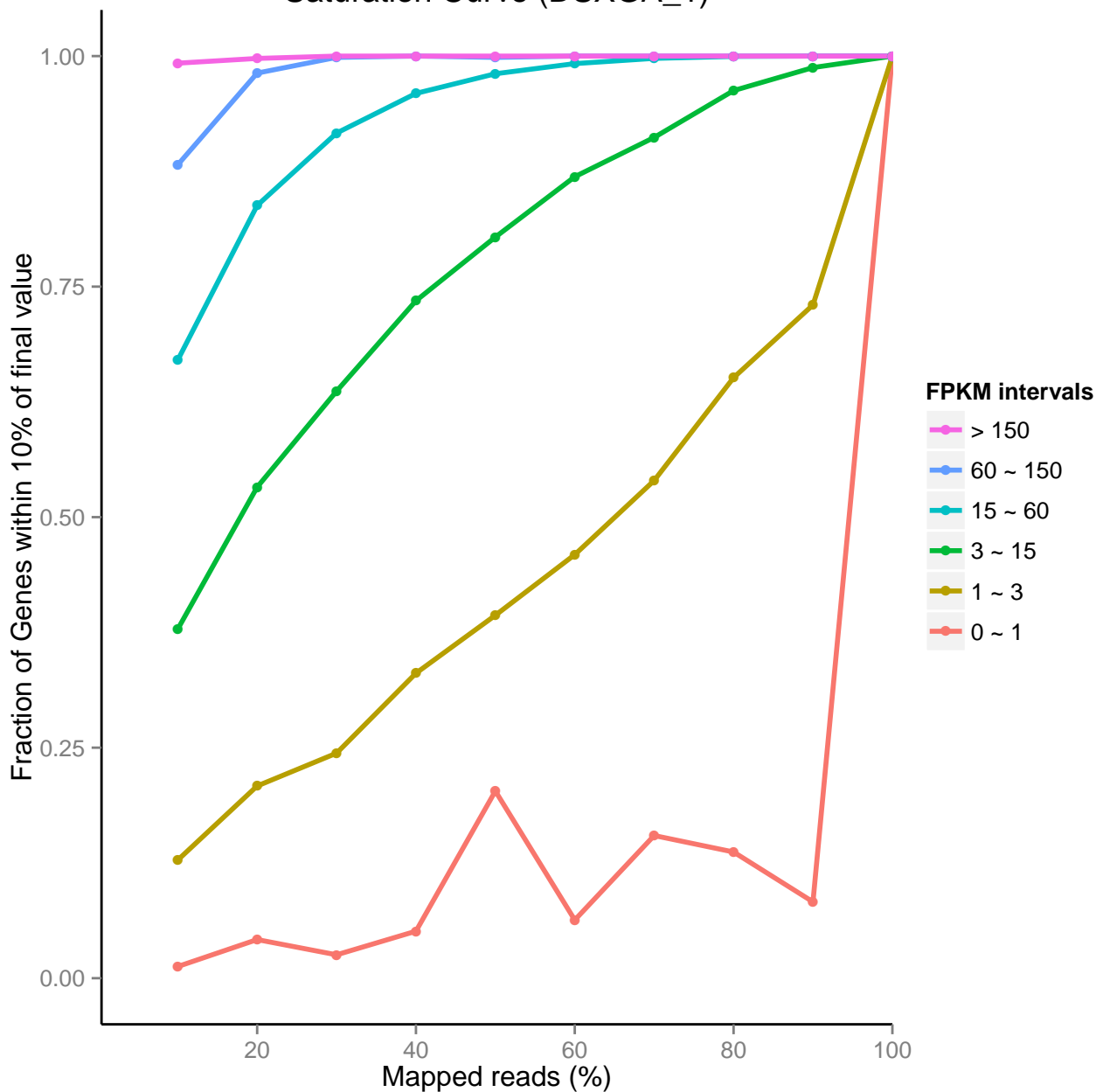

Supplement: Supplementary file 11 — This file provides the saturation curves of the gene expression levels of G. yamadae and G. asiaticum. (ZIP 2739 kb) [file 12864_2017_4059_MOESM11_ESM.zip › Additional file 11/GYA/DSXGA_1.Saturation_curve.pdf]

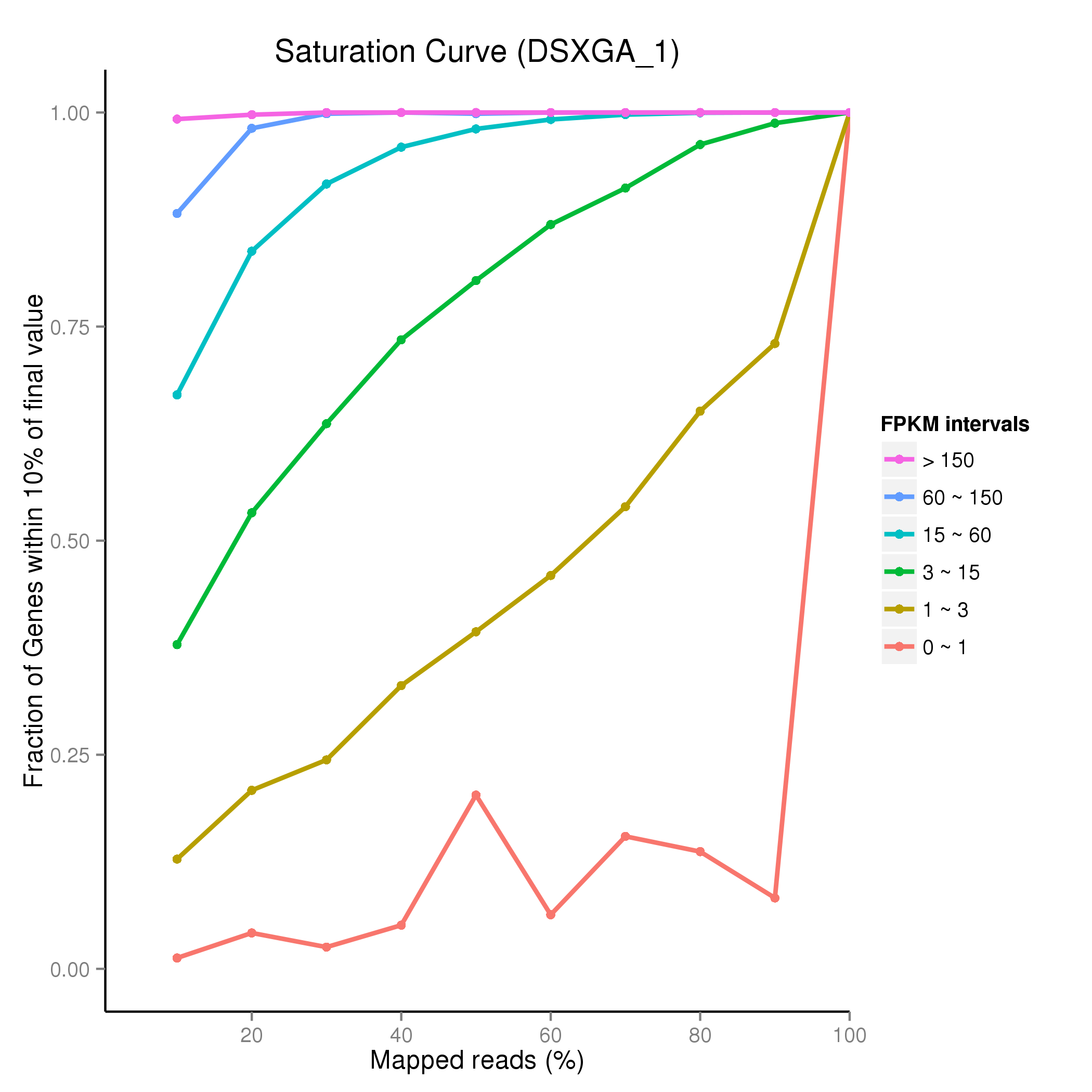

Supplement: Supplementary file 11 — This file provides the saturation curves of the gene expression levels of G. yamadae and G. asiaticum. (ZIP 2739 kb) [file 12864_2017_4059_MOESM11_ESM.zip › Additional file 11/GYA/DSXGA_1.Saturation_curve.png]

Saturation Curve (DSXGA\_2)

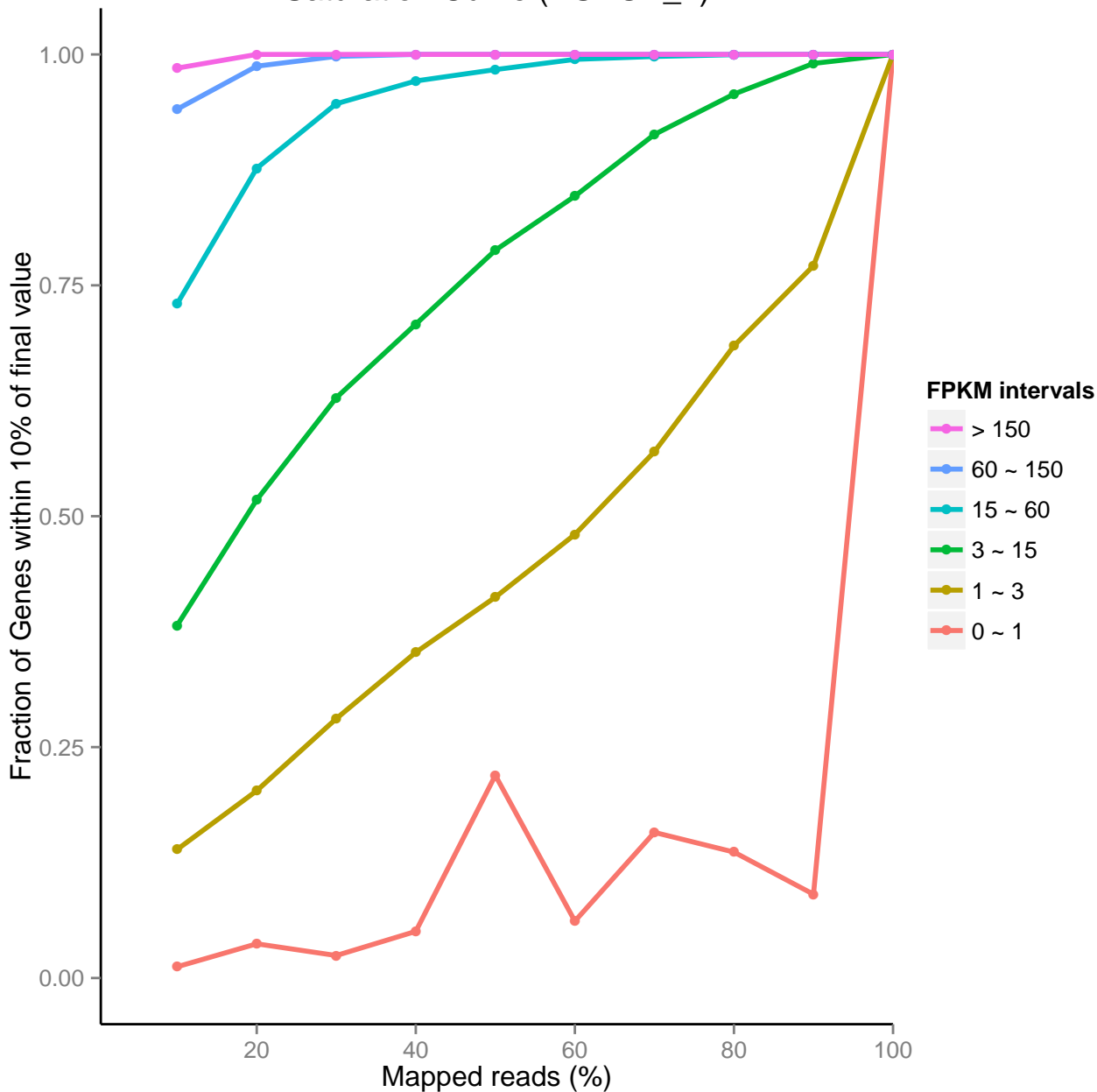

Supplement: Supplementary file 11 — This file provides the saturation curves of the gene expression levels of G. yamadae and G. asiaticum. (ZIP 2739 kb) [file 12864_2017_4059_MOESM11_ESM.zip › Additional file 11/GYA/DSXGA_2.Saturation_curve.pdf]

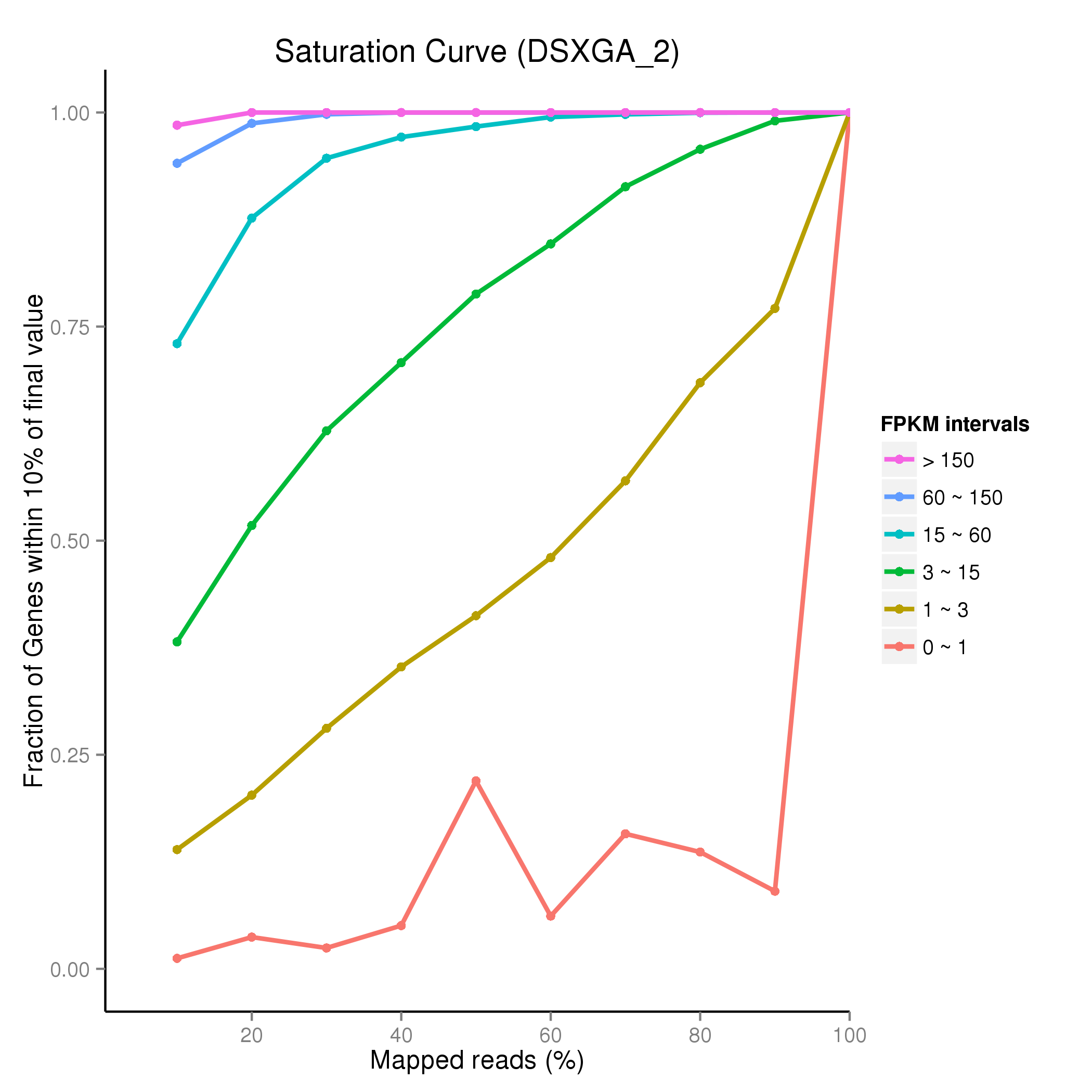

Supplement: Supplementary file 11 — This file provides the saturation curves of the gene expression levels of G. yamadae and G. asiaticum. (ZIP 2739 kb) [file 12864_2017_4059_MOESM11_ESM.zip › Additional file 11/GYA/DSXGA_2.Saturation_curve.png]

Saturation Curve (DSXGA\_3)

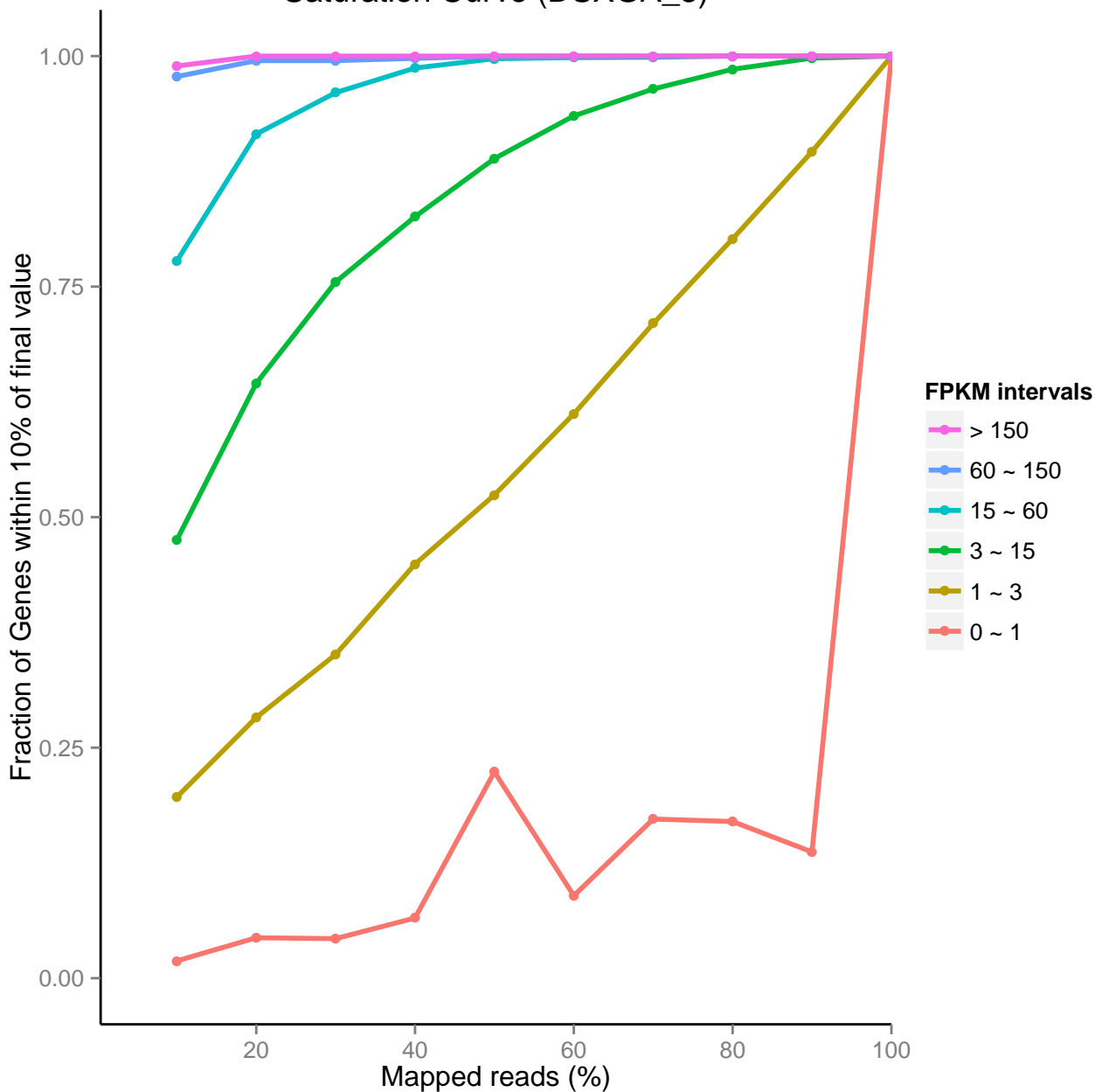

Supplement: Supplementary file 11 — This file provides the saturation curves of the gene expression levels of G. yamadae and G. asiaticum. (ZIP 2739 kb) [file 12864_2017_4059_MOESM11_ESM.zip › Additional file 11/GYA/DSXGA_3.Saturation_curve.pdf]

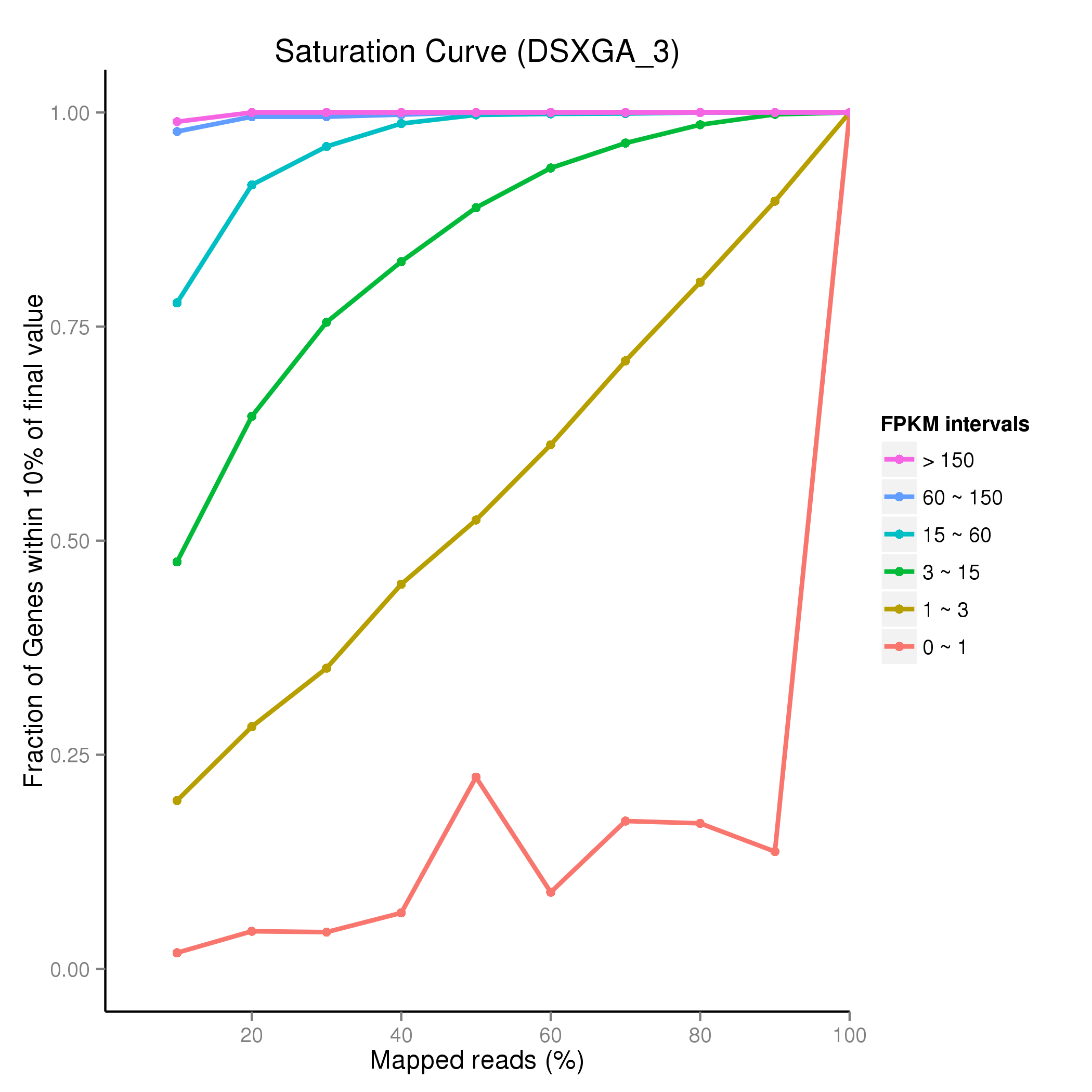

Supplement: Supplementary file 11 — This file provides the saturation curves of the gene expression levels of G. yamadae and G. asiaticum. (ZIP 2739 kb) [file 12864_2017_4059_MOESM11_ESM.zip › Additional file 11/GYA/DSXGA_3.Saturation_curve.png]

Saturation Curve (DSXGY\_1)

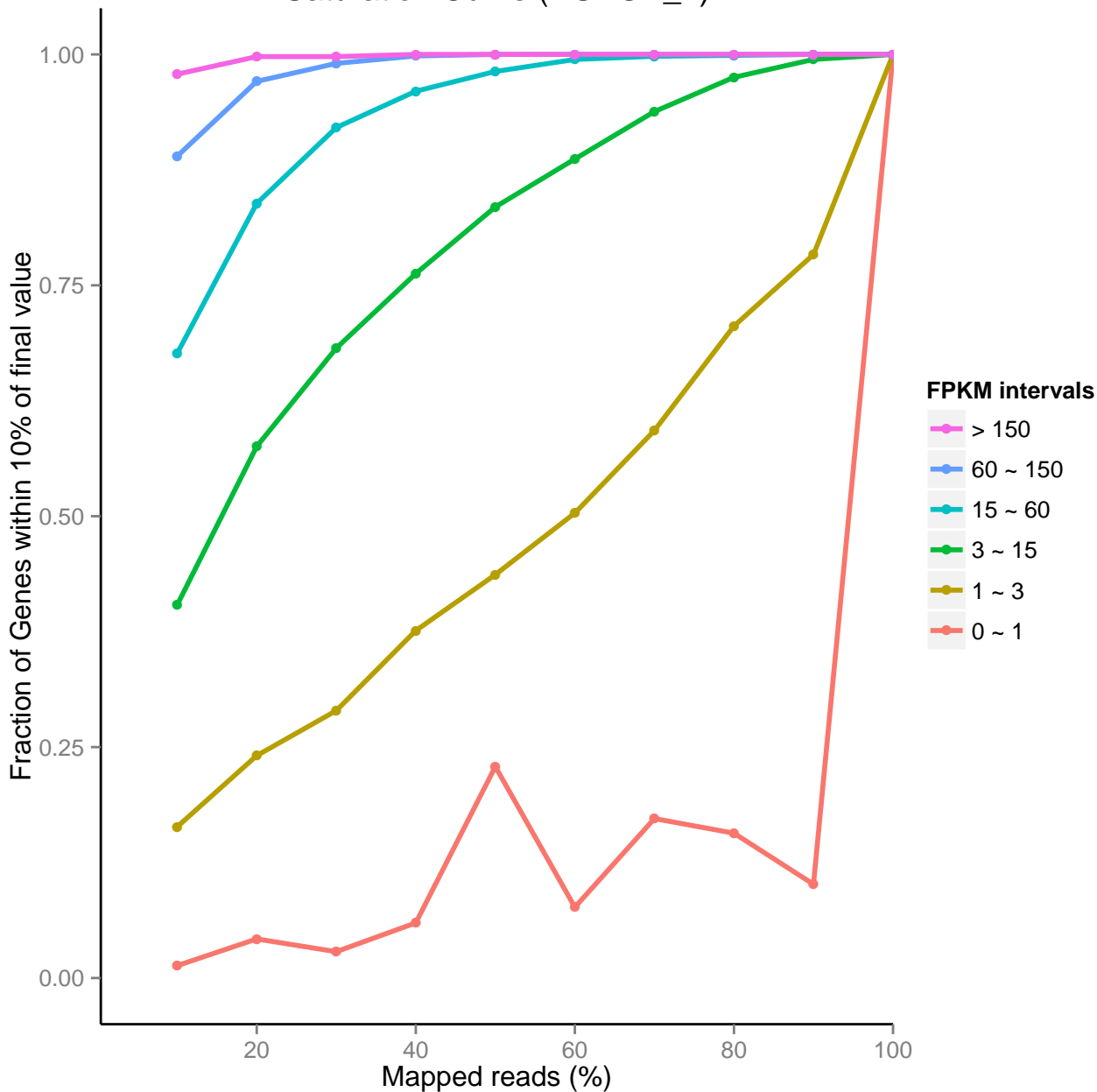

Supplement: Supplementary file 11 — This file provides the saturation curves of the gene expression levels of G. yamadae and G. asiaticum. (ZIP 2739 kb) [file 12864_2017_4059_MOESM11_ESM.zip › Additional file 11/GYY/DSXGY_1.Saturation_curve.pdf]

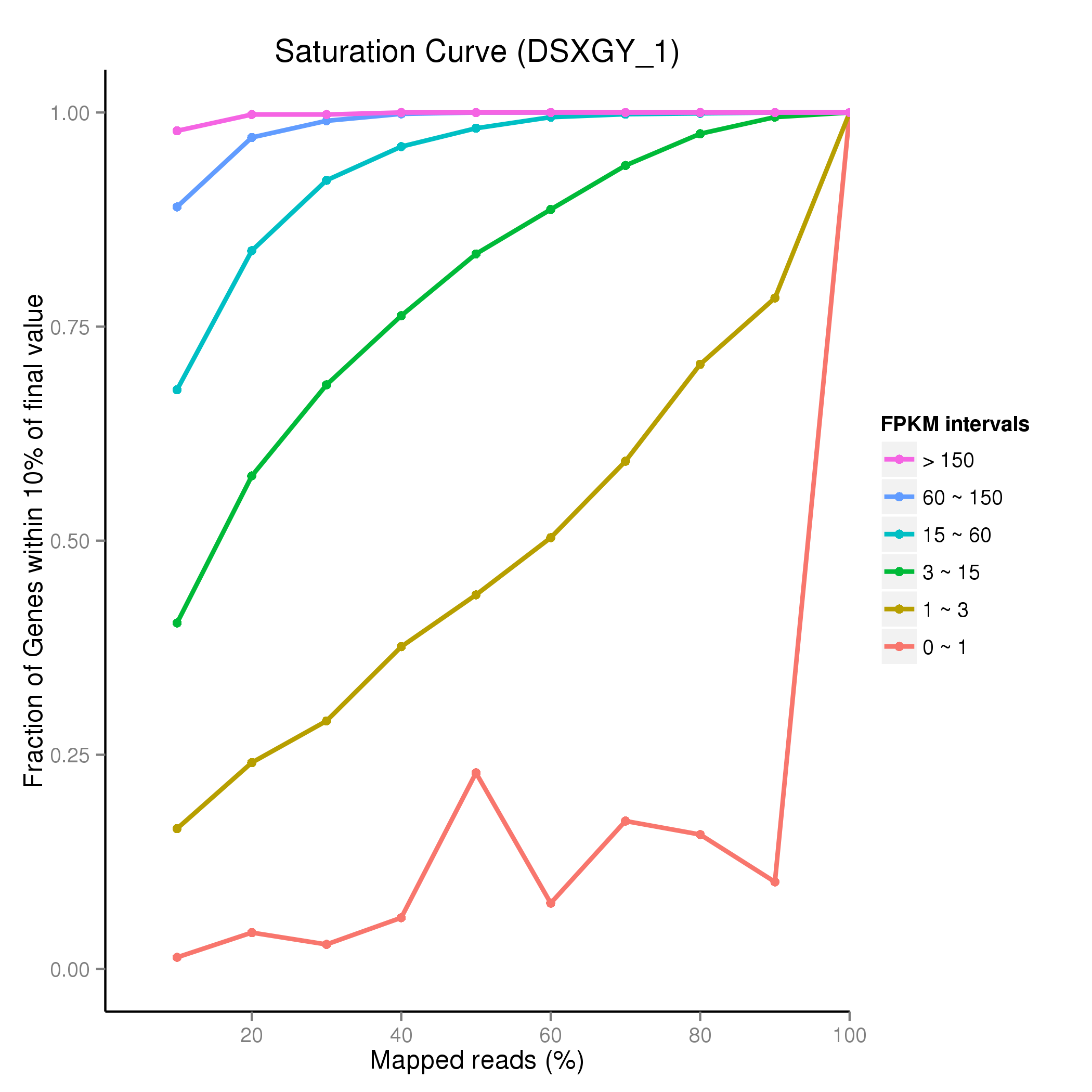

Supplement: Supplementary file 11 — This file provides the saturation curves of the gene expression levels of G. yamadae and G. asiaticum. (ZIP 2739 kb) [file 12864_2017_4059_MOESM11_ESM.zip › Additional file 11/GYY/DSXGY_1.Saturation_curve.png]

Saturation Curve (DSXGY\_2)

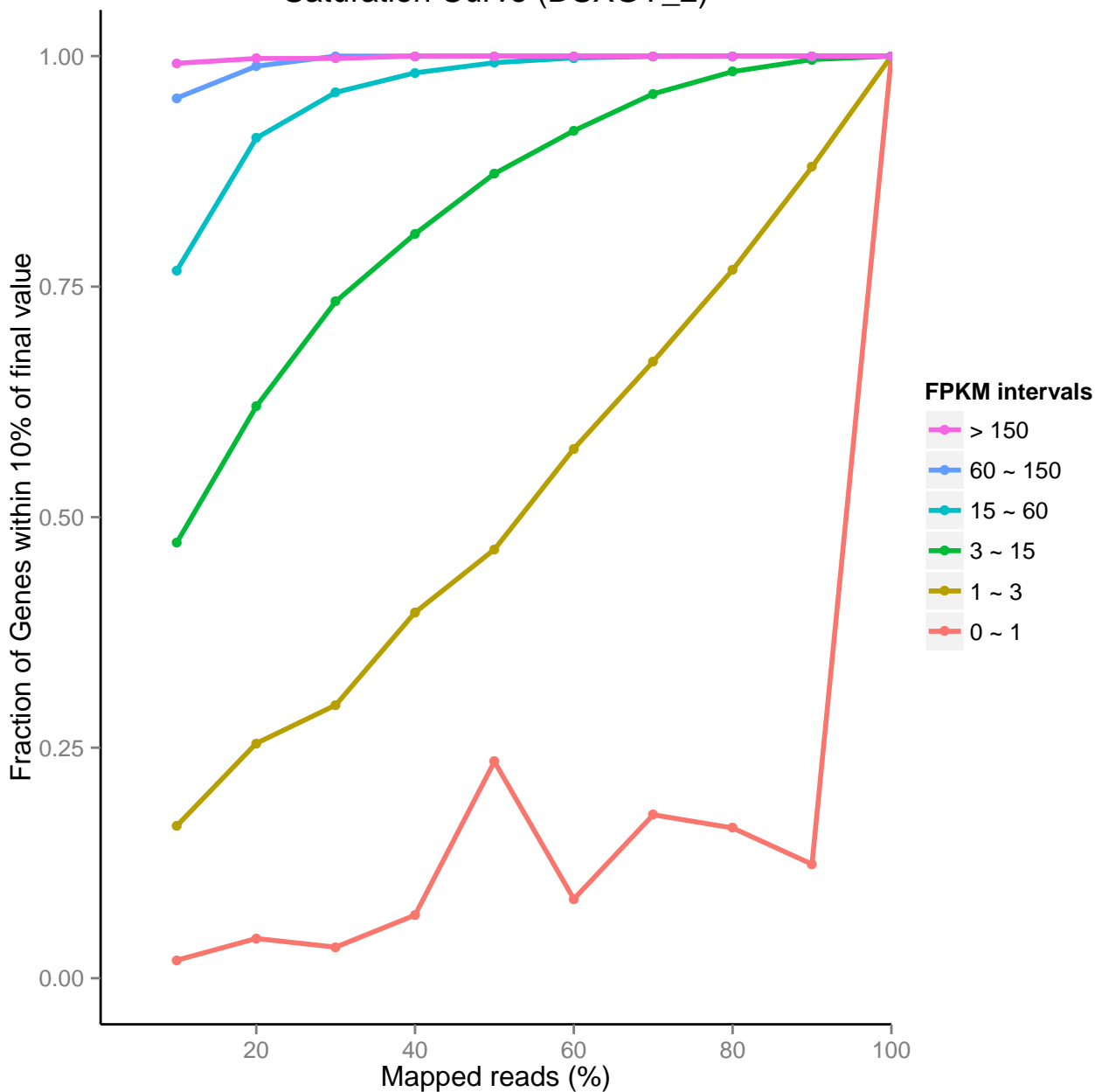

Supplement: Supplementary file 11 — This file provides the saturation curves of the gene expression levels of G. yamadae and G. asiaticum. (ZIP 2739 kb) [file 12864_2017_4059_MOESM11_ESM.zip › Additional file 11/GYY/DSXGY_2.Saturation_curve.pdf]

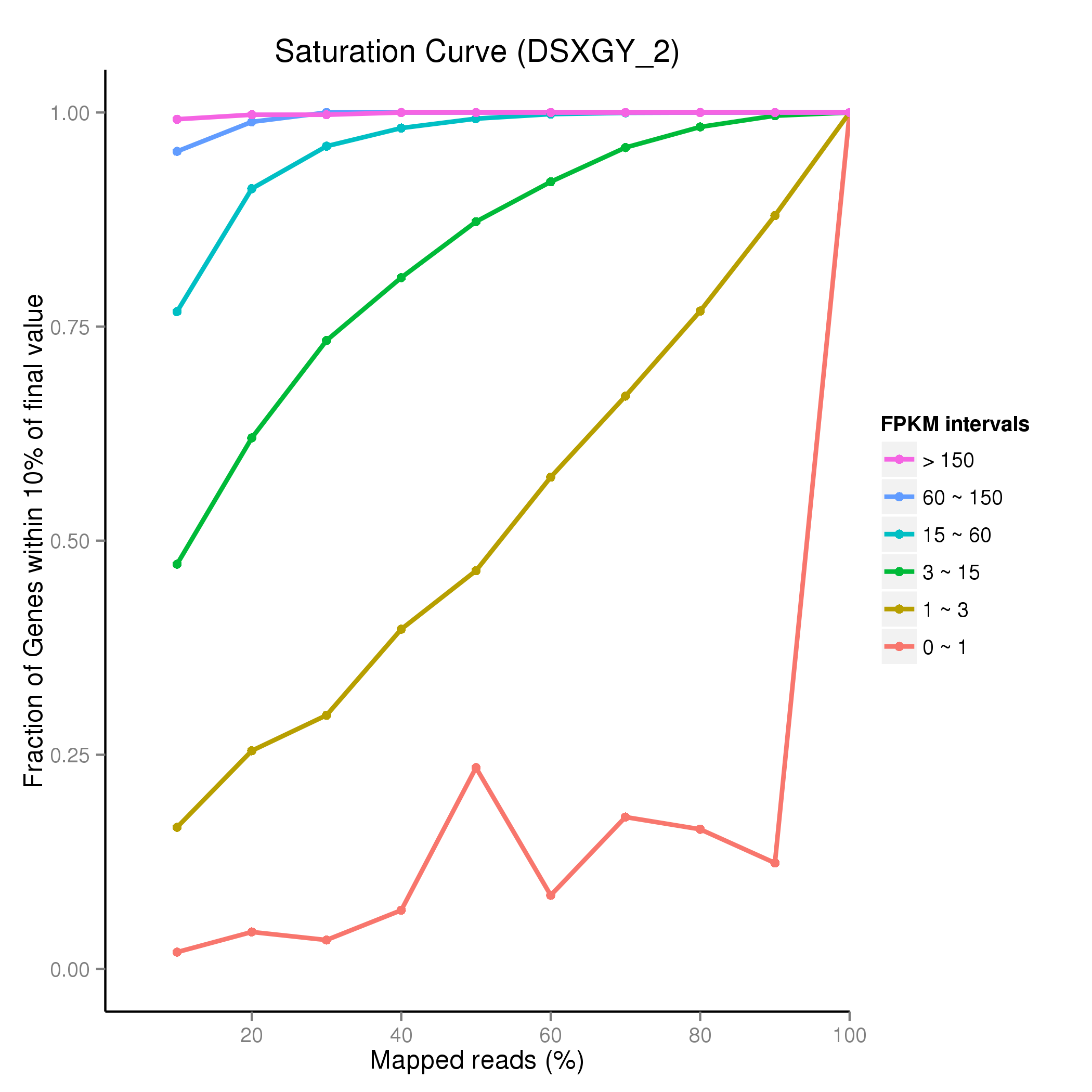

Supplement: Supplementary file 11 — This file provides the saturation curves of the gene expression levels of G. yamadae and G. asiaticum. (ZIP 2739 kb) [file 12864_2017_4059_MOESM11_ESM.zip › Additional file 11/GYY/DSXGY_2.Saturation_curve.png]

Saturation Curve (DSXGY\_3)

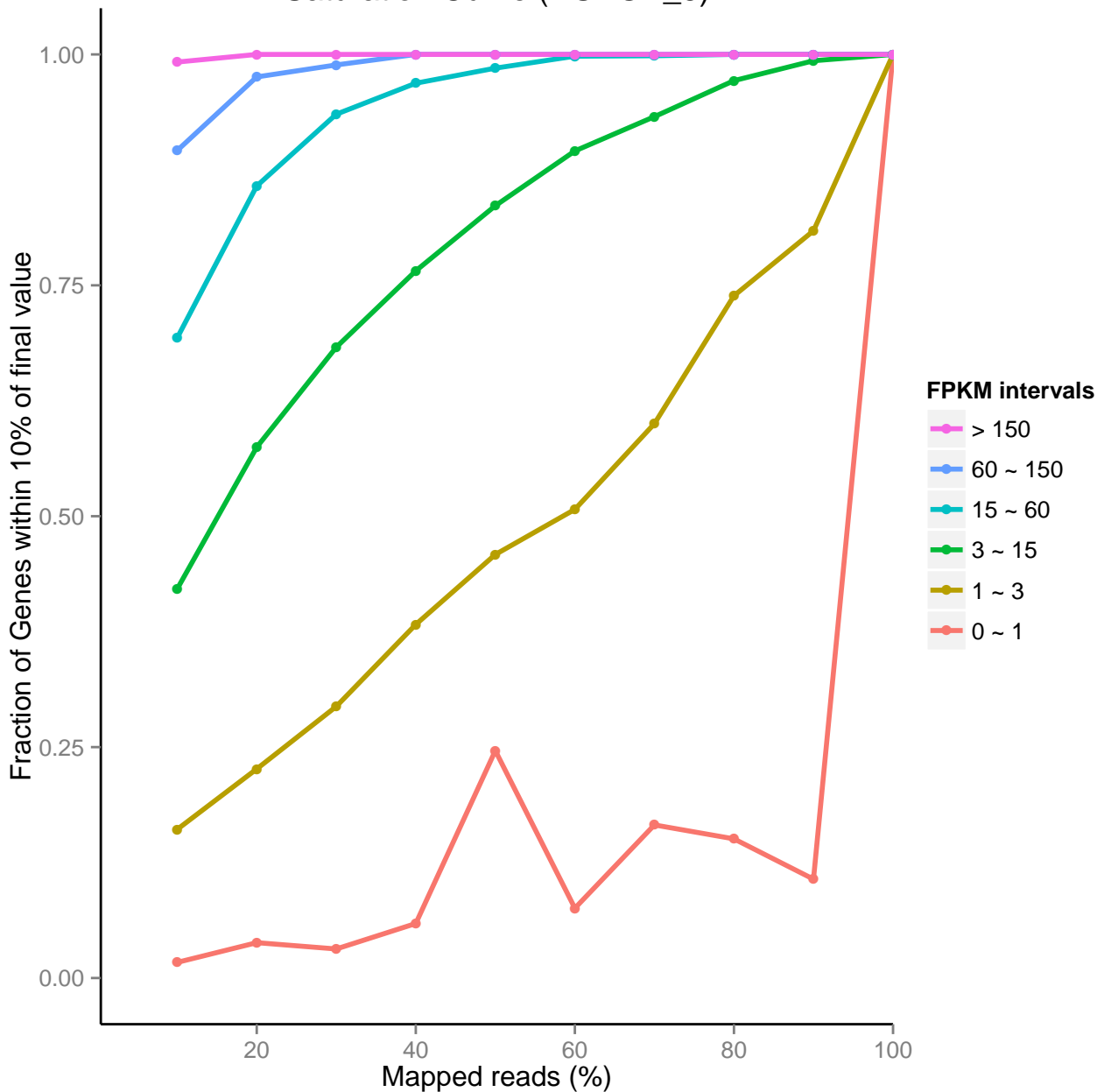

Supplement: Supplementary file 11 — This file provides the saturation curves of the gene expression levels of G. yamadae and G. asiaticum. (ZIP 2739 kb) [file 12864_2017_4059_MOESM11_ESM.zip › Additional file 11/GYY/DSXGY_3.Saturation_curve.pdf]

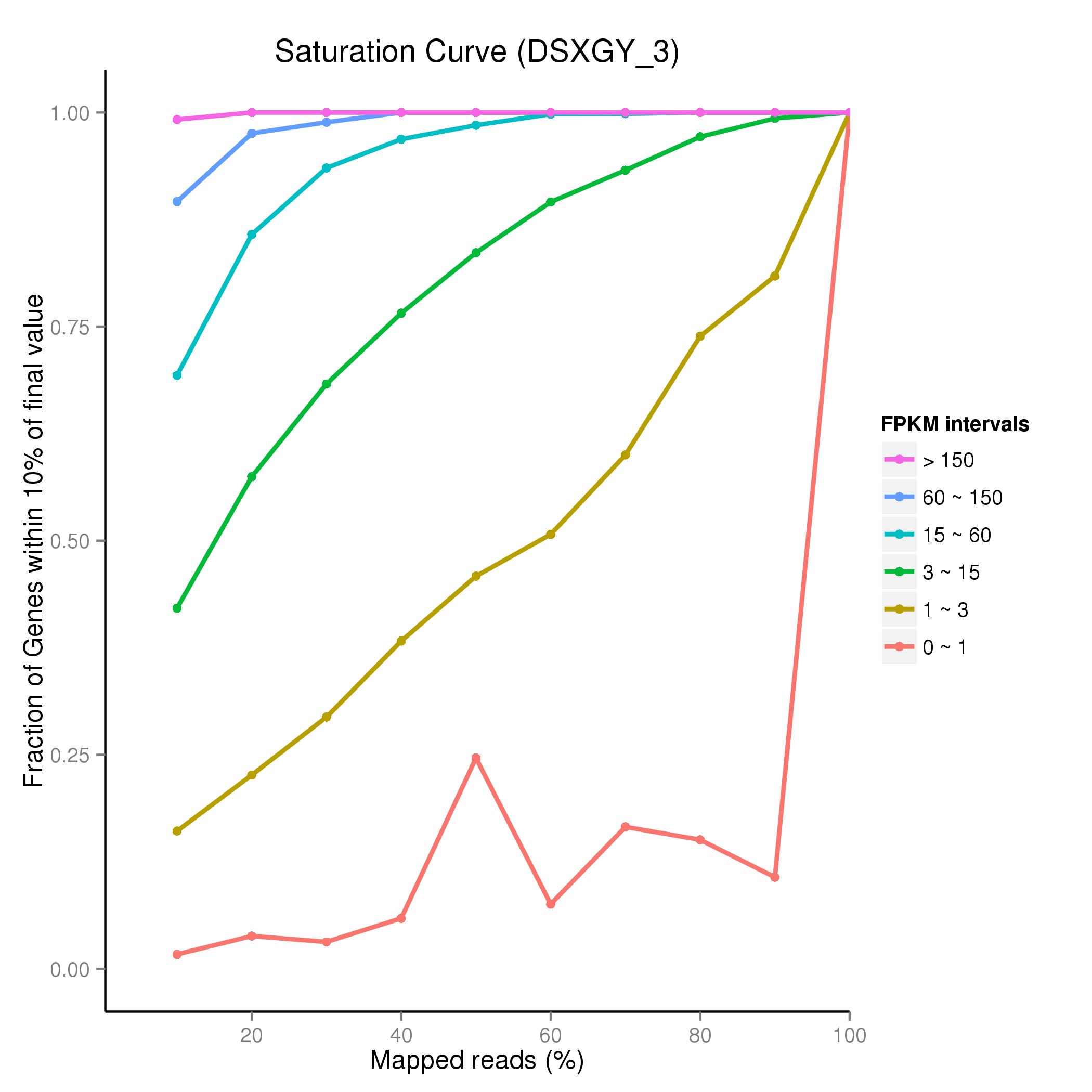

Supplement: Supplementary file 11 — This file provides the saturation curves of the gene expression levels of G. yamadae and G. asiaticum. (ZIP 2739 kb) [file 12864_2017_4059_MOESM11_ESM.zip › Additional file 11/GYY/DSXGY_3.Saturation_curve.png]
